# Impact of London's low emission zone on air quality and children's respiratory health: a sequential annual cross-sectional study

**DOI:** 10.1016/S2468-2667(18)30202-0

**Published:** 2018-11-15

**Authors:** Ian S Mudway, Isobel Dundas, Helen E Wood, Nadine Marlin, Jeenath B Jamaludin, Stephen A Bremner, Louise Cross, Andrew Grieve, Alex Nanzer, Ben M Barratt, Sean Beevers, David Dajnak, Gary W Fuller, Anna Font, Grainne Colligan, Aziz Sheikh, Robert Walton, Jonathan Grigg, Frank J Kelly, Tak H Lee, Chris J Griffiths

**Affiliations:** a Medical Research Council (MRC)–Public Health England Centre for Environmental Health, National Institute for Health Research Biomedical Research Centre at Guy's and St Thomas' National Health Service Foundation Trust and King's College London, London, UK; b Asthma UK Centre for Applied Research, Barts Institute of Population Health Sciences, Queen Mary University of London, London, UK; c Institute for Research in Molecular Medicine (INFORMM), Universiti Sains Malaysia, Health Campus, Kelantan, Malaysia; d MRC and Asthma UK Centre in Allergic Mechanisms of Asthma, King's College London, London, UK; e Asthma UK Centre for Applied Research, Centre for Medical Informatics, Usher Institute of Population Health Sciences and Informatics, The University of Edinburgh, Edinburgh, UK; f Allergy Centre, HK Sanatorium and Hospital, Hong Kong Special Administrative Region, China

## Abstract

**Background:**

Low emission zones (LEZ) are an increasingly common, but unevaluated, intervention aimed at improving urban air quality and public health. We investigated the impact of London's LEZ on air quality and children's respiratory health.

**Methods:**

We did a sequential annual cross-sectional study of 2164 children aged 8–9 years attending primary schools between 2009–10 and 2013–14 in central London, UK, following the introduction of London's LEZ in February, 2008. We examined the association between modelled pollutant exposures of nitrogen oxides (including nitrogen dioxide [NO_2_]) and particulate matter with a diameter of less than 2·5 μm (PM_2·5_) and less than 10 μm (PM_10_) and lung function: postbronchodilator forced expiratory volume in 1 s (FEV_1_, primary outcome), forced vital capacity (FVC), and respiratory or allergic symptoms. We assigned annual exposures by each child's home and school address, as well as spatially resolved estimates for the 3 h (0600–0900 h), 24 h, and 7 days before each child's assessment, to isolate long-term from short-term effects.

**Findings:**

The percentage of children living at addresses exceeding the EU limit value for annual NO_2_ (40 μg/m^3^) fell from 99% (444/450) in 2009 to 34% (150/441) in 2013. Over this period, we identified a reduction in NO_2_ at both roadside (median −1·35 μg/m^3^ per year; 95% CI −2·09 to −0·61; p=0·0004) and background locations (−0·97; −1·56 to −0·38; p=0·0013), but not for PM_10_. The effect on PM_2·5_ was equivocal. We found no association between postbronchodilator FEV_1_ and annual residential pollutant attributions. By contrast, FVC was inversely correlated with annual NO_2_ (−0·0023 L/μg per m^3^; −0·0044 to −0·0002; p=0·033) and PM_10_ (−0·0090 L/μg per m^3^; −0·0175 to −0·0005; p=0·038).

**Interpretation:**

Within London's LEZ, a smaller lung volume in children was associated with higher annual air pollutant exposures. We found no evidence of a reduction in the proportion of children with small lungs over this period, despite small improvements in air quality in highly polluted urban areas during the implementation of London's LEZ. Interventions that deliver larger reductions in emissions might yield improvements in children's health.

**Funding:**

National Institute for Health Research Biomedical Research Centre at Guy's and St Thomas' National Health Service (NHS) Foundation Trust and King's College London, NHS Hackney, Lee Him donation, and Felicity Wilde Charitable Trust.

## Introduction

Air pollution is a leading cause of global mortality, with WHO estimates of 7 million premature deaths partly attributable to air pollution in 2012, which is approximately one in eight global deaths.^1^ Of these, 3·7 million relate to outdoor air pollution. With increasing population growth and urbanisation, air quality has emerged as an important determinant of public health within cities,^2^, ^3^, ^4^ with the health burden falling disproportionately on disadvantaged populations who are less able to choose the environments in which they live.^5^ Primary studies and systematic reviews have linked air pollution with adverse effects across the lifecourse,^6^ from increased risk of preterm birth^7^ and incident childhood asthma,^8^ to premature cardiopulmonary mortality.^9^ Although urban air pollution reflects contributions from a range of local and regional sources, studies and reviews have highlighted the importance of traffic-related air pollutants on a range of health endpoints, with a particular focus on the contribution of diesel emissions to poor air quality in Europe.^6^, ^10^, ^11^, ^12^

Childhood and adolescence are periods of rapid growth during which organ systems are particularly susceptible to injury.^13^ The ESCAPE meta-analysis of data for 5921 children from five European birth cohorts showed that poor air quality was associated with reduced lung function in preadolescent children (aged 6–8 years).^14^ Adolescents (aged 9–14 years) showed clinically important restrictions on lung growth and function in the southern California (USA) Children's Health Study.^15^, ^16^ Even in relatively low pollution environments (Stockholm County, Sweden), lung growth in adolescents (aged 16 years) has been related to early life pollutant exposures.^17^ Impaired lung development in childhood has impacts that carry into adulthood, with morbidity and mortality linked to reduced adult lung function.^18^ A causal linkage between air pollution exposure and suboptimal lung growth has been further supported by analysis of consecutive longitudinal cohorts in the Children's Health Study,^19^ in which the proportion of children with clinically small lungs was reduced as air quality improved between 1994 and 2011. These data strongly suggest that policies designed to reduce air pollution can deliver a measurable health benefit.

**Research in context**

**Evidence before this study**

Exposure to traffic pollutants has been associated with adverse health effects, especially in children, with the ESCAPE meta-analysis of data for 5921 children showing that poor air quality was associated with reduced lung function in preadolescent children (aged 6–8 years). The introduction of low emission zones (LEZ) has been proposed to improve air quality and improve public health. These operate either by restricting vehicle entry into urban areas, or through fixed penalties on polluting vehicles to encourage uptake of lower emission technologies. Despite the political and financial costs of LEZ implementation, studies on the impact of these schemes on air quality and public health remain scarce. We searched MEDLINE for publications from 2009 until Nov 1, 2017, using keywords “low emission zone” AND/OR “traffic”, “air quality”, and “health”. These reviews showed LEZs do not consistently improve air quality, and effects are small. Few studies addressed health impacts, and these have tended to rely on modelled predictions of air quality improvements.

**Added value of this study**

We exploited the comprehensive monitoring network in London, UK, to evaluate the changes in air pollution following the introduction of the LEZ. Our study covered the tightening of emission controls within the LEZ in 2012, which occurred alongside national, regional, and local policies to improve air quality. We based our study in four inner-city London boroughs, which were identified as non-compliant with EU annual NO_2_ limit values at the start of study. Despite the problems associated with vehicle non-compliance with European class emission standards over this period, we have provided evidence of improvements in air quality. We also confirmed the previously reported association between pollutant exposures and reduced children's lung volume. We observed some evidence of a reduction in rhinitis, but not asthma symptoms or the proportion of children with small lungs over the study period.

**Implications of all available evidence**

Large-scale LEZs can deliver improvements in urban air quality and these can be linked to changes in childhood respiratory health. However, more ambitious schemes than those evaluated here are required to meet legislative limits and deliver improvements to respiratory health in many European cities.

Low emission zones (LEZ), areas where the entry of polluting vehicles is restricted or penalised on the basis of European class emission standards, are often used as the major component of emission control strategies. London, UK, introduced the world's largest citywide LEZ in 2008 (appendix 1 p 4). Across Europe, about 200 LEZs are now in operation, with others in use in Asia, including Singapore and Tokyo. Despite the widespread application of LEZs to improve air quality, evidence that LEZs reduce pollutant concentrations or improve health is scarce.^20^, ^21^, ^22^, ^23^, ^24^, ^25^, ^26^, ^27^ Holman and colleagues^26^ concluded that LEZs in operation in European countries had inconsistent effects on PM_10_ and nitrogen dioxide (NO_2_) concentrations. Wang and colleagues^28^ found only six studies that addressed impact of traffic LEZ-type emission control interventions on health.^29^, ^30^, ^31^, ^32^, ^33^ Of these, only one^31^ gathered health data directly from individuals, finding negligible effects on respiratory symptoms. The other studies relied on modeling the effects of predicted (not necessarily achieved) emission reductions on health. Health equity has been assessed in only two studies, with opposing conclusions.^20^, ^30^

The phased introduction of London's LEZ, beginning in 2008, provided the opportunity for a natural experiment to evaluate the effect of this emission-based mitigation strategy. A description of the various phases of the LEZ is presented in appendix 1 (p 4), with the early phases in 2008 (phases 1 and 2), followed by a further tightening of emission controls in 2012 (phase 3). Our objective in this study was to evaluate its effect by examining the association between pollutant exposures and respiratory health in school children living within highly polluted areas of central London over the period 2009–14. Our aim was to test the hypothesis that improvements in air quality would be associated with improved respiratory health. We focused on children living in areas of London not meeting the EU NO_2_ limit value (annual mean of 40 μg/m^3^), over a period in which improvements in air quality were predicted to occur because of improved emission controls on diesel powered heavy and light goods vehicles.

## Methods

### Study design and study population

We used a sequential cross-sectional study to avoid risks of attrition in a mobile inner-city population with a classic cohort design. We invited 28 primary schools in the London boroughs of Tower Hamlets, Hackney, Greenwich, and the City of London to participate, focusing on schools close to air quality monitoring stations to maximise the accuracy of exposure data, reflecting a range of distances from major roads (<500 m). Within these schools, all children in year 4 (aged 8–9 years) were invited to participate; we had no exclusion criteria. Data were collected during winter periods: from November 2008, to January 2009 (pilot study), and between November and March or April for the full 5 year study (2009–10 until 2013–14). The pilot was used to evaluate the feasibility of doing bronchodilator lung function tests within the school setting and establish the required sample size for the full study (2009–14; years 1–5). Information about the study was sent home in school bags with each child, along with a consent form and a questionnaire for parents to complete and return to the school. During a single study visit to each school each year, health assessments were done to examine lung function and collect biological samples (appendix 1 p 5). Additional information on sex, age, ethnicity, and residential address was obtained from school records. We assigned socioeconomic status according to the residential address using the Index of Multiple Deprivation (IMD). Height and weight were measured during the health assessment and the body-mass index (BMI) calculated.

### Ethics

Parents gave written consent and children verbal assent, to participate in the health assessment. The study was approved by the local research ethics committee (East London and The City HA Local Research Ethics Committee [REC] 2, REC ref number 08-H0704-139) and conformed to the standards set by the Declaration of Helsinki.

### Long-term and short-term exposure attributions

We estimated exposures at the residential and school address from annual (2008–13) nitrogen oxides (NO_x_), NO_2_ particulate matter with a diameter of less than 10 μm (PM_10_), and less than 2·5 μm (PM_2·5_) maps of London using the KCLurban model with the Atmospheric Dispersion Modelling System dispersion model version 4 and road source model version 2.3 (Cambridge Environmental Research Consultants 19),^34^ measured hourly meteorological data, and empirically derived NO–NO_2_–O_3_ and PM associations and emissions from the London Atmospheric Emissions Inventory (appendix 1 p 7).^35^ In this analysis, we also weighted these annual exposure estimates for periods spent at the home and school address points, based on the following criteria: 84·4% of time at home and 15·6% at school, based on a 7 h school day for 5 days per week, 39 weeks per year. Thus, each child's weighted exposure was estimated by the addition of time at home multiplied by 0·884 with time at school multiplied by 0·156.

We derived acute exposure estimates at the address point by scaling annual mean concentrations according to a nowcast factor (appendix 1 pp 8–10) calculated for each pollutant for periods immediately before lung function evaluation. This factor is defined as the ratio between concentrations, measured by a local subset of continuous air pollution monitoring sites in the previous period and the annual mean of measurements at the same sites. For this study, we calculated nowcast scaling factors for the 3 h period immediately before the school day (0600–0900 h), 24 h, and 7 days before the school visits to reflect both acute and subchronic exposure periods. To derive NO_x_ and NO_2_, we averaged measurements of scaling factors across 14–17 urban background and roadside sites within and surrounding the London boroughs of Tower Hamlets and Hackney, on the basis of data availability. For the PM_10_ and PM_2·5_ scaling factors, measurements from 9–13 and 14–20 background and roadside sites were averaged, respectively. We have summarised the correlations between pollutant attributions at each exposure interval in appendix 1 (p 11–14) for each study year, including the pilot.

### Lung function assessments

Trained investigators assessed children's respiratory function by spirometry (MicroLab, Micro Medical, Carefusion, Wokingham, UK), according to American Thoracic Society (ATS) and European Respiratory Society (ERS) guidelines^36^ with baseline and postbronchodilator measurements, following administration of salbutamol 400 μg by large volume spacer. Each spirometry assessment aimed to obtain three acceptable and two repeatable attempts both before and after bronchodilator. Quality control was based on the ATS and ERS guidelines, modified for children. Spirometry data were quality controlled by three senior respiratory scientists, with one performing this role throughout the full 6 year duration of the study (appendix 1 p 15).

### Symptoms

We collected information on respiratory and allergic symptoms using a parent-completed questionnaire, based on the validated International Study of Asthma and Allergies in Childhood (ISAAC) questionnaire, as described previously,^27^ and current and lifetime symptoms were defined (appendix 1 p 16). Information on the questionnaires was entered as recorded, regardless of apparent inconsistencies. Unanswered questions were coded as missing. We calculated symptom prevalence by dividing the number of positive responses by the total number of completed questionnaires, as described previously.^27^

### Urine collection and analysis

Spot urine samples were collected for the determination of cotinine concentrations using a commercial microplate enzyme immunoassay (Cozart Forensic Microplate EIA for cotinine; M155B1; Concateno, Abingdon, UK). Urinary creatinine concentrations were also measured with a commercially available kit (Cayman Chemical Company, Ann Arbor, MI, USA). Exposure to environmental tobacco smoke was defined as a creatinine-corrected cotinine value greater than 30 ng/mg.

### Outcomes

The study was powered for the primary objective: a 4% year-on-year improvement in forced expiratory volume in 1 s (FEV_1_) in year 4 school children living within London's LEZ. Secondary objectives were assessing improvement in other lung function parameters: forced vital capacity (FVC) and a reduction in respiratory symptoms, by use of the modified ISAAC questionnaire.

### Evaluation of air quality changes over the study period

To evaluate air pollution trends, we calculated running annual mean concentrations (2006–14) of the main criterion pollutants (NO_2_, PM_10_, and PM_2·5_), plus NO_x_, for inner and outer London roadside and background locations, using a subset of the London Air Quality Network monitoring sites within and surrounding the study area (appendix 2). Additionally, for the period of the LEZ (2008–13), we calculated linear trends using the Theil-Sen estimator method, from monthly means, previously deseasonalised with the seasonal and trend decomposition technique, Loess.^37^ Forest plots were then produced for those sites having at least a 75% capture rate over the 5 year period, beginning 2008 and ending 2013, and the overall trend was calculated by fitting the linear random-effects model DerSimonian-Laird estimator, as previously described.^38^

### Statistical analysis and sample size calculations

We established the required sample size from data collected during the pilot year (2008–09). These pilot data showed the mean FEV_1_ to be 1·71 L (SD 0·28), with valid measurements obtained from 150 (74%) of 202 children. To detect a 4% increase in FEV_1_ from 1·71 L to 1·78 L in two successive years with 80% power at a 5% significance level would require 245 children per year under simple random sampling. Assuming 74% of children returned valid lung function measurements, the mean cluster size for analysis would be 22 children out of a class of 30 children. The design effect, based on an intracluster correlation coefficient of 0·03, was:

1+(22-1)×0·03=1·63

which inflated the sample size to 400. Therefore, 19 classes (rounded up from 18·2; calculated by 400 divided by 22) were needed to be sampled in the full study.

We used linear mixed models with a random effect for school to examine the effects of children's air pollutant exposures, over prescribed intervals (0600–0900 h on the day of the school visit, 24 h before, 7 days before, and the annual values) on lung function outcomes, including a sensitivity analysis using prebronchodilator values. We selected baseline characteristics a priori to be included in the models: age, sex, height, BMI, self-reported ethnicity (Asian, black, white, or mixed or other), socioeconomic status, and exposure to environmental tobacco smoke. To assess the assumption of no change over time, the study year was also included as a covariate in the models.

Respiratory and allergic symptoms were recoded as binary variables and their associations with annual air pollution exposures, adjusted for the covariates outlined above, were evaluated with mixed-effects logistic regression. Study years were also included in the model to account for any year-on-year changes. All statistical evaluations were based on the data from study years 1–5, and the impact of including the data from the pilot year was also examined as a sensitivity analysis. Furthermore, as this study was based on postbronchodilator lung function, we also examined the associations with prebronchodilator baseline lung function to allow comparison with previous studies with this approach only. We used multi-pollutant models, but because of the high correlation between the four selected pollutants at each sampling interval across the study years (appendix 1 pp 12–14) they were not informative. Statistical significance was assumed at the 5% level. All statistical analyses were done with Stata version 14.1.

### Role of the funding source

The funders had no role in the design, execution, analysis, interpretation, or writing of the study. ISM, HEW, NM, SAB, and CJG had access to the raw data. The corresponding author had full access to all of the data and the final responsibility to submit for publication.

## Results

**Figure 1:**
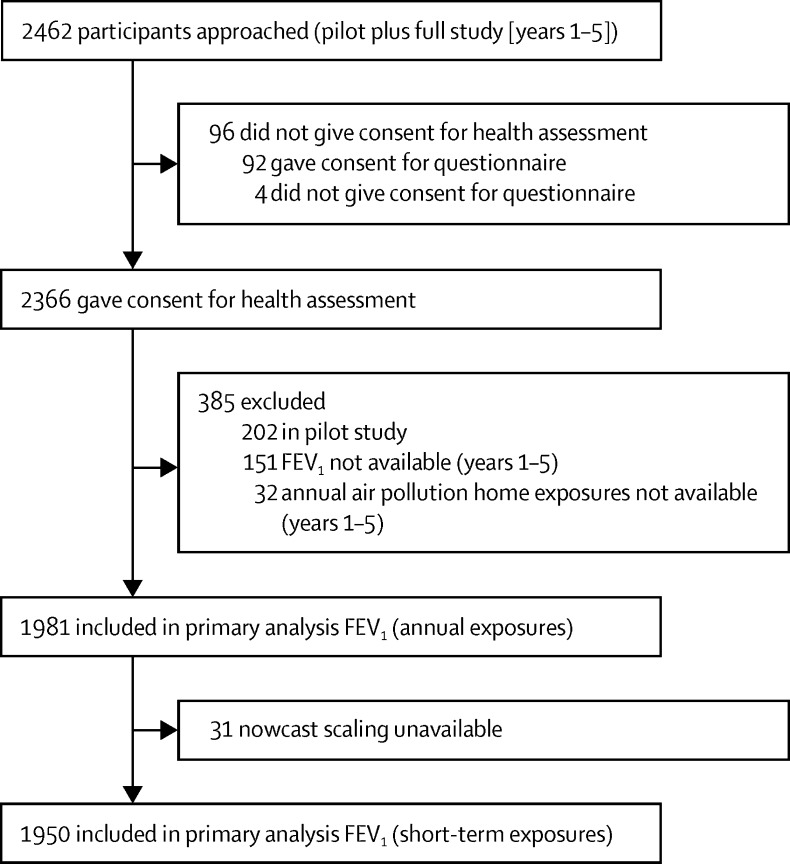
Study flowchart FEV_1_=forced expiratory volume in 1 s.

Across the full study (years 1–5) and the pilot study, 2462 children were approached, of whom 2366 (96%) consented to the full health assessment (figure 1). Of the 2164 children recruited into the full study running from the winter period from 2009–10 to 2013–14, the primary outcome—technically acceptable FEV_1_—was obtained from 2013 children (mean FEV_1_ of 1·69 L; mean FVC of 1·87 L for 1969 children; appendix 1 p 17). Of these participants, annual air pollution attributions were available for 1981, with nowcast-adjusted acute exposure estimates available for 1950 children (figure 1). Overall, there appeared to be slightly more girls than boys, with the largest ethnic group being Asian (table). Environmental tobacco smoke exposure, assessed as a urinary cotinine concentration of more than 30 mg/mg creatinine, was observed in 15% (332/2164) of the cohort, but fell from 21% (94/441) in year 1 to 11% (50/438) in year 5 (table). A comparison of the demographic and exposure data for the children included (n=1981) and excluded (n=183) from the primary analysis is included in appendix 1 (p 18). This comparison shows that no systematic bias was introduced through the exclusion of this group of children. Additionally, key demographic details of the study boroughs over years 1–5 of the study are shown in appendix 1 (p 19), showing the representativeness of participants in relation to the demography of study boroughs. The associations between the demographic variables with lung function are summarised in appendix 1 (p 20). For both FEV_1_ and FVC, significant associations were observed with age, gender, height, and BMI, but not with IMD score, or evidence of environmental tobacco smoke exposure (appendix 1 p 20). Clear differences in lung function variables between the three major ethnic groups were observed, with the Asian population (the largest) as the comparator. Overall, white children's FEV_1_ and FVC were larger than those of Asian children, which in turn were larger than those reported in black children (appendix 1 p 20). After controlling for all annual pollutant exposures, lung function parameters were not different between study years.

**Table:** Participant demographics by study year

		**Pilot (2008–09; N=202)**	**Year 1 (2009–10; N=441)**	**Year 2 (2010–11; N=418)**	**Year 3 (2011–12; N=436)**	**Year 4 (2012–13; N=431)**	**Year 5 (2013–14; N=438)**	**Years 1–5 (2009–14; N=2164)**
Demographics								
	Age (years)	8·8 (0·3)	8·8 (0·3)	8·8 (0·3)	8·9 (0·3)	8·9 (0·3)	8·8 (0·3)	8·9 (0·3)
	Height (cm)	133·7 (6·3; N=192)	133·8 (7·0; N=416)	133·7 (6·6; N=405)	134·2 (6·6; N=423)	133·9 (6·9; N=420)	134·0 (6·8; N=427)	133·9 (6·8; N=2091)
	Weight (kg)	32·3 (7·4; N=192)	32·7 (8·3; N=416)	32·4 (7·8; N=404)	32·5 (7·8; N=423)	32·3 (7·8; N=420)	32·5 (8·0; N=426)	32·5 (7·9; N=2089)
	BMI (kg/m^2^)	17·9 (3·0; N=192)	18·1 (3·3; N=416)	17·9 (3·3; N=404)	17·9 (3·3; N=423)	17·8 (3·3; N=420)	18·0 (3·3; N=426)	17·9 (3·3; N=2089)
	IMD score	46·3 (11·3; N=201)	45·7 (10·0; N=441)	44·7 (12·0; N=416)	43·7 (12·0; N=428)	46·4 (10·6; N=423)	44·6 (12·0; N=432)	45·0 (11·4; N=2140)
Sex								
	Male	112 (55%)	227 (51%)	201 (48%)	175 (40%)	201 (47%)	209 (48%)	1013 (47%)
	Female	90 (45%)	211 (48%)	215 (51%)	261 (60%)	230 (53%)	229 (52%)	1146 (53%)
	Not reported	0	3 (1%)	2 (<1%)	0	0	0	5 (<1%)
Environmental tobacco smoke exposure								
	>30 ng/mg	58 (29%)	94 (21%)	66 (16%)	56 (13%)	66 (15%)	50 (11%)	332 (15%)
	≤30 ng/mg	118 (58%)	302 (68%)	332 (79%)	357 (82%)	347 (81%)	361 (82%)	1699 (79%)
	Not reported	26 (13%)	45 (10%)	20 (5%)	23 (5%)	18 (4%)	27 (6%)	133 (6%)
Children living at addresses not meeting the EU annual limit value for NO_2_ (>40 μg/m^3^)*		130/199 (65%)	444/450 (99%)	342/459 (75%)	90/431 (21%)	302/440 (69%)	150/441 (34%)	1458/2420 (60%)
Reported ethnicity								
	Asian	77 (38%)	162 (37%)	144 (34%)	157 (36%)	188 (44%)	169 (39%)	820 (38%)
	Black	50 (25%)	110 (25%)	103 (25%)	101 (23%)	93 (22%)	108 (25%)	515 (24%)
	White	59 (29%)	124 (28%)	107 (26%)	112 (26%)	85 (20%)	100 (23%)	528 (24%)
	Mixed or other	16 (8%)	44 (10%)	63 (15%)	66 (15%)	62 (14%)	58 (13%)	293 (14%)
	Not reported	0	1 (<1%)	1 (<1%)	0	3 (1%)	3 (1%)	8 (<1%)

Data are mean (SD), mean (SD; N), n (%), or n/N (%). BMI=body-mass index. IMD=Index of Multiple Deprivation.

* Based on all participants for whom linked modelled data were available.

**Figure 2:**
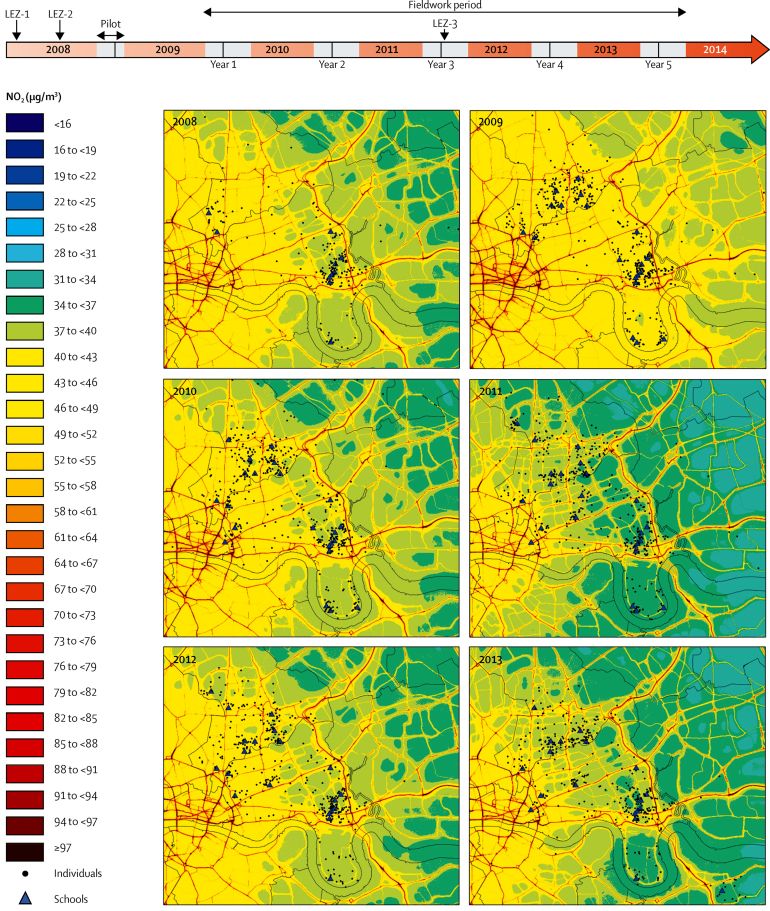
Study timeline and annual NO_2_ models The upper panel shows the study timeline relative to the various phases of the London low emission zone (appendix 1 p 4). The shaded areas represent the annual collection windows, which ran over the winter periods. The lower panels represent the annual NO_2_ pollution maps (2008–13) used for the exposure assessments. NO_2_=nitrogen dioxide. LEZ=London emission zone.

**Figure 3:**
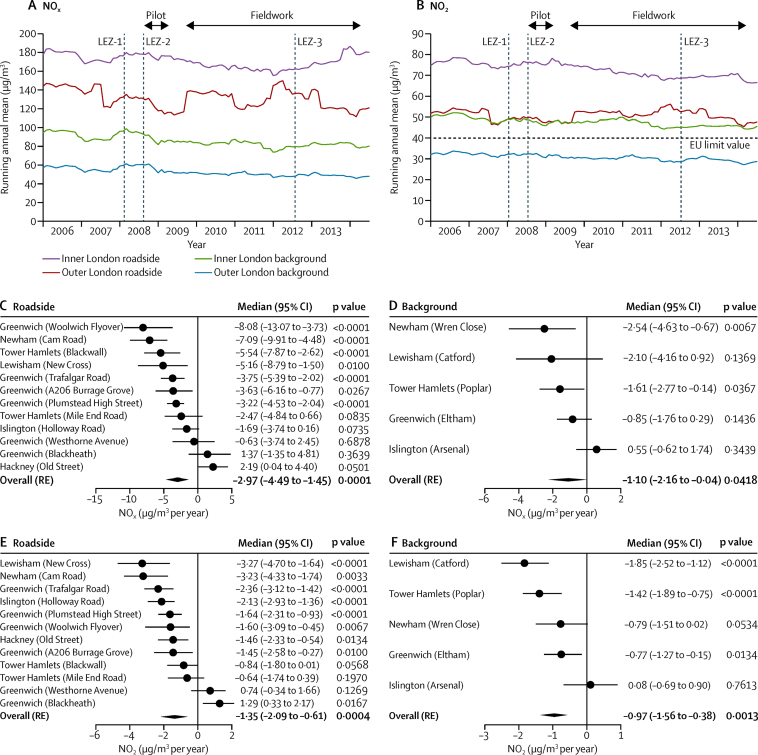
Running annual mean NO_x_ (A) and NO_2_ (B) concentrations at London roadside and background sites from 2006 to 2014 Background sites are within and surrounding the study area. Air pollution is shown relative to the three phases of the LEZ (appendix 1 p 4). Forest plots for roadside and background NO_x_ (C, D) and NO_2_ (E, F) across the period 2008–13 by site and aggregated across sites (RE, refers to the overall trend). Data are presented as the median of the slopes between all pairs of points from the monthly mean concentration time series; 95% CIs were calculated by bootstrap sampling. NO_x_=nitrogen oxides. NO_2_=nitrogen dioxide. LEZ=London emission zone. RE=random effect.

Annual maps for NO_2_ (figure 2), NO_x_ (appendix 1 p 21), PM_10_ (appendix p 22), and PM_2·5_ (appendix 1 p 23) are illustrated with the attributed median and 25th and 27th quartiles exposures for the study participants summarised in appendix 1 (p 24). Although the individual annual exposure attributions over the 5 years of the study were broadly equivalent (eg, median of 40·7 μg/m^3^ [IQR 38·7–43·3] for NO_2_; appendix 1 p 24), examination of the running annual mean concentrations for NO_2_ (figure 3B) and PM_2·5_ (appendix 1 p 25) from a selection of monitoring sites within and surrounding the study area over the period from the start of 2006, to mid-2014, provided some evidence of a downward trend, most markedly for NO_2_ at the inner London roadside (from a mean annual concentration of 77·15 μg/m^3^ [SD 1·24] in 2006, to 69·76 μg/m^3^ [0·47] in 2013). These illustrative time trends provide no evidence of clear step changes in pollutant concentrations following the various phases of the LEZ (figure 3, appendix 1 p 24). To examine changes in air quality in greater detail, linear trends over the period 2008–13 were calculated with the Theil-Sen method for a number of roadside and background sites. For both NO_x_ and NO_2_, despite considerable heterogeneity between sites, evidence was clear for a decrease in both roadside (median −2·97 μg/m^3^ per year for NO_x_; 95% CI −4·49 to −1·45; p=0·0001; and −1·35 μg/m^3^ per year for NO_2_; −2·09 to −0·61; p=0·0004) and urban background concentrations (−1·10; −2·16 to −0·04; p=0·0418; and −0·97; −0·97 to −0·38; p=0·0013; respectively; figure 3C–F). For NO_2_, this result corresponded to an overall reduction of 6·75 μg/m^3^ for roadside locations and 4·85 μg/m^3^ at background locations over the 5 year study period. Concordant with this observation, the proportion of children living at addresses exceeding the EU limit value for NO_2_ fell across the study period, from 99% (444/450) in study year 1 to 34% (150/441) in study year 5, based on all children for whom modelled estimates were available (table). The outcome for PM_10_ and PM_2·5_ was less clear, with no evident reduction at roadside sites (appendix 1 p 25).

**Figure 4:**
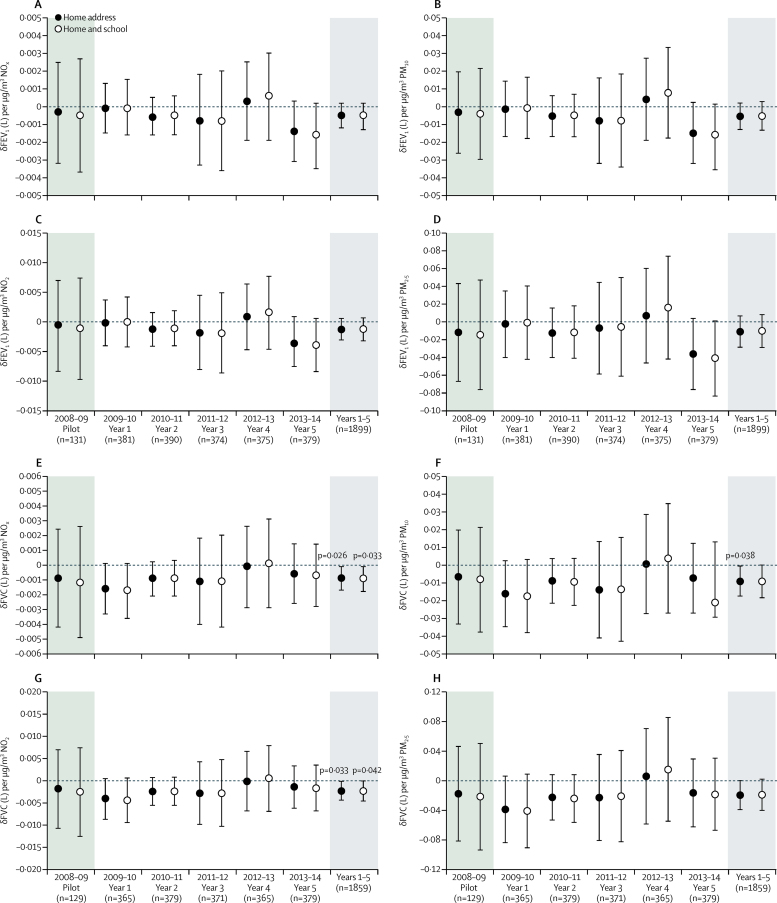
Change in FEV_1_ (A–D) and FVC (E–H) per unit change in NO_x_, NO_2_, PM_10_ and PM_2·5_ annual concentrations Analyses were based on residential address or were weighted for periods spent at home and school addresses. Data are mean (95% CI) for each study year, plus the pilot, and aggregated across study years 1–5. FEV_1_=forced expiratory volume in 1 s. NO_x_=nitrogen oxides. NO_2_=nitrogen dioxide. PM_10_=particulate matter with a diameter of less than 10 μm. PM_2·5_=particulate matter with a diameter of less than 2·5 μm.

**Figure 5:**
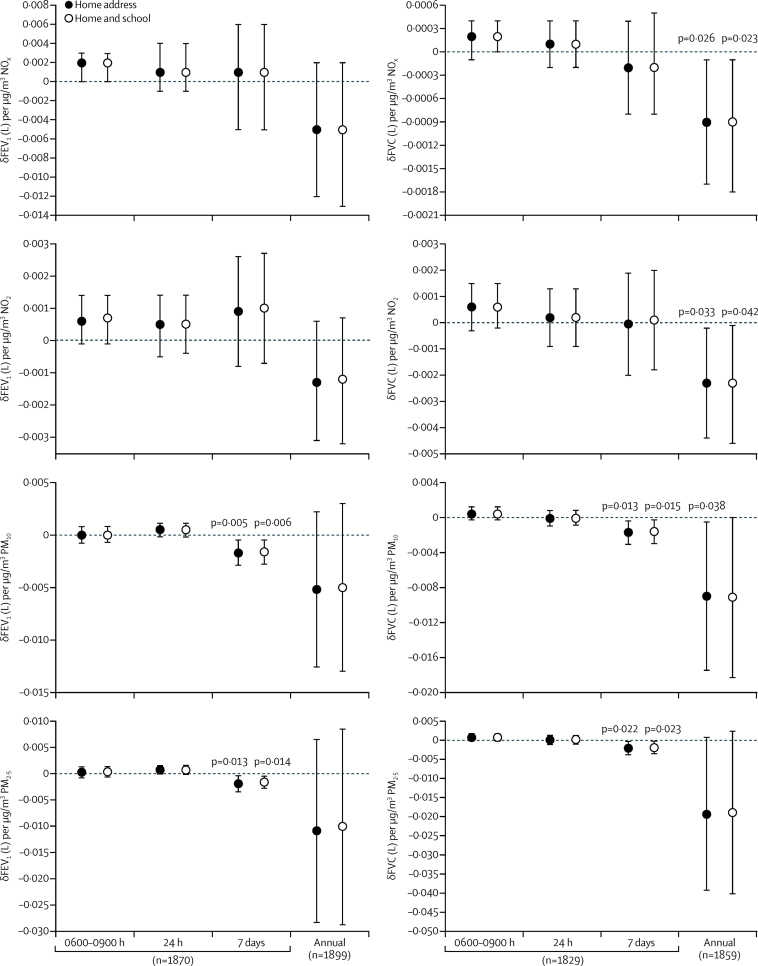
Associations of the four selected pollutants with FEV_1_ and FVC, with 3 h (0600–0900 h), 24 h, 7 day, and annual exposure attributions Analyses were based on residential address and were weighted for periods spent at school. Data are presented as mean (95% CI) pooled across years 1–5. FEV_1_=forced expiratory volume in 1 s. FVC=forced vital capacity. NO_x_=nitrogen oxides. NO_2_=nitrogen dioxide. PM_10_=particulate matter with a diameter of less than 10 μm. PM_2·5_=particulate matter with a diameter of less than 2·5 μm.

We analysed the associations between postbronchodilator FEV_1_ (figure 4A–D) and FVC (figure 4E–H), by year and aggregated across study years 1–5. For FEV_1_, associations were small with confidence intervals that spanned zero and did not attain statistical significance (figure 4). Inclusion of the data collected in the study pilot did not alter this outcome (appendix 1 p 26). By contrast, FVC was inversely associated with annual NO_x_, NO_2_ (−0·0023 L/μg per m^3^; −0·0044 to −0·0002; p=0·033), and PM_10_ (−0·0090 L/μg per m^3^; −0·0175 to −0·0005; p=0·038) exposures (figure 4E–H, appendix 1 p 27), with no clear evidence of an impact of study year. Neither FEV_1_ nor FVC were associated with exposures on the morning of the assessment (0600–0900 h), or in the previous 24 h, but robust negative associations were observed in the 7 day average PM_10_ and PM_2·5_ exposures (figure 5). We have provided data as change in volume (L) per μg/m^3^ of pollutant exposure (Figure 4, Figure 5) and also as IQR annual exposures for FEV_1_ and FVC (appendix 1 p 28) for both home and home and school exposure attributions. As many previous studies examining air pollution lung function associations have used lung function without bronchodilation, we ran this investigation as a post-hoc sensitivity analysis (appendix 1 p 29). Although the associations between annual pollutant exposure and FVC were attenuated and did not attain statistical significance (p values ranged 0·050 to 0·072, based on home exposures), the data revealed similar trends. The inverse association between 7 day average PM_10_ and PM_2·5_ with reduced FEV_1_ and FVC were robust to the use of prebronchodilator values (appendix 1 p 29). Despite the evidence of reductions in roadside and background NO_2_ and the association between annual pollutants exposures and reduced FVC, we observed no clear reduction in the proportion of children with predicted lung function less than 80%, 85%, or 90% of predicted (appendix 1 p 30). We found limited evidence of significant associations between current and lifetime respiratory and allergic symptoms with annual pollutant attributions (appendix 1 p 31). Of the symptoms examined, only current rhinitis symptoms showed a positive association with the annual pollutant concentrations, although these did not attain statistical significance (eg, p=0·09 for PM_10_). When data from the pilot year were included in these analyses the association with PM_10_ was significant (p=0·010), as was the association with PM_2·5_ (p=0·017), consistent with earlier observations on children living within London.^27^ We therefore examined the yearly associations in current rhinitis and lifetime asthma (appendix 1 p 32). This analysis indicated that the prevalence of rhinitis fell markedly from the period of the pilot and the first year of the study onward (appendix 1 p 32).

## Discussion

Over the period covering the implementation of London's LEZ, we identified evidence of reduced NO_2_ and NO_x_ concentrations at roadside and background locations within the study area, in the absence of improvements in PM_10_. The proportion of children living at locations that did not meet the EU annual limit value for NO_2_ (40 μg/m^3^) fell threefold over the same period, although there was considerable inter-year variation. Importantly, these measured changes in pollutant concentrations occurred in parallel to published evidence of good compliance with the tighter emission standards.^39^ Furthermore, there was no evidence that the compliance within London resulted in a displacement of more polluting vehicles to regions outside London's LEZ.^39^ Despite these improvements, we observed evidence of reduced lung volumes in children associated with NO_x_, NO_2_, and PM_10_ annual exposures over the same period, with no evidence of a decrease in the proportion of children with small lung volumes for their age over the 5 year study. The associations with NO_x_ and NO_2_ were independent of shorter-term exposure estimates, suggesting that the reduced volumes reflected the longer-term impact of air pollution. Notably, despite evidence of significant improvements in air quality over the duration of the study, considerable areas of inner and outer London remain above the EU NO_2_ limit value (figure 2). Although we found evidence for improvements at roadside and background sites, at the current rate of change, full compliance with EU limit values for NO_2_ for London remains distant, without a substantial tightening of current emission controls.

The main strengths of our study included the intensity of both traffic exposures and coverage of the available air quality monitoring, including measurement sites specifically established to monitor the impact of London's LEZ. Our study took place in an area where a substantial proportion of children live in areas that do not meet EU targets for NO_2_ exposure, with most schools and residences all within 500 m of busy roads. Our data, from 2009–10 onwards, cover a period when traffic fleets have become increasingly dominated by diesel vehicles, with associated problems of high NO_2_ concentrations and primary particle emissions. Consequently, we believe that our study is unique in reflecting the impact of a modern European city's air quality environment. A key innovation of our study was the use of novel modelling of exposures that allowed us to differentiate effects of short-term, medium-term, and long-term exposures on the same spatial scale. Our use of postbronchodilator values provided reliable estimates of children's lung capacities and therefore high-quality lung function data. Concerns about attrition in an urban mobile population meant that we chose not to use a classic longitudinal cohort design. This choice restricted our capacity to directly address lung growth or to quantify improvements associated with improved air quality. Our study also lacked a control population that was not subject to the effects of the LEZ, which was a weakness, and our focus on inner-city boroughs restricted the range of exposure contrasts that would have been achieved by a broader geographical coverage. For NO_2_ and PM_10_, this outcome was less of a problem as the exposure ranges were still large, but for PM_2·5_ we had relatively little exposure contrast. Additionally, as our study did not commence until after the introduction of the initial phases of the LEZ in February, 2008, we have probably underestimated the full effects of the scheme.

The ESCAPE project examined the association between lung function data collected from children (aged 6–8 years) across five European cohorts, between 2002 and 2007, with annual pollutant exposures based on residential address using land use regression models. Furthermore, the effects of short-term exposures on the lung function measurements were assessed based on measurements made at regional and urban background monitoring sites. This analysis, with a combined population of 5921 children, showed annual exposures to NO_2_, NO_x_, PM_10_, and PM_2·5_ were associated with significant reductions in FEV_1_.^14^ The negative impact of primary traffic-derived pollutants on children's lung function has also been further reinforced by two US studies, in which traffic exposures were associated with reduced FEV_1_ and FVC,^15^, ^16^ and in the Children's Health Study,^19^ which showed that reductions in pollution delivered clinically meaningful improvements in FEV_1_ and FVC.

Our study confirms and extends these observations, by showing inverse associations between lung function and exposure to urban air, particularly to NO_x_ and NO_2_, which are good proxies for diesel emissions within London. As lung function decrements have been observed in response to experimental diesel challenges^40^ and real-world exposures to air pollution at high diesel traffic locations,^41^ we had to establish that acute exposures were not affecting the results observed. We therefore derived short-term exposure estimates for varying periods running up to each child's health assessment using the nowcast method. This analysis showed that the morning and 24 h exposures before the health assessments had little impact on children's lung function, reinforcing the view that the association between the annual exposure and reduced FVC was indicative of a chronic impact on lung function, most probably explained by reduced lung growth in this cohort. Of note, compared with these studies, the annual exposures to pollutants in our London cohort, particularly for NO_x_ and NO_2_, are very high. For example, they are greater than the NO_2_ concentrations in Urman and colleagues' study^42^ of high pollution communities, and almost two-fold higher than the concentrations in the highest exposure cohort in the ESCAPE project^14^ (GINI and LISA cohorts): mean 43·52 μg/m^3^ (SD 5·45) versus 23·4 μg/m^3^ (2·8). The range of NO_2_ exposures observed within the Swedish BAMSE cohort, the largest of the ESCAPE children's cohorts (44% of the total population) with the lowest exposures, varied between 6·0 μg/m^3^ and 33·0 μg/m^3^.^14^ Therefore, all the annual NO_2_ exposure attributions within that cohort were below the range observed in London: 31·2–98·9 μg/m^3^. The higher concentrations observed in our study reflect not only the more urban nature of the study population, but also the increased concentrations of NO_2_ and NO_x_ observed within European cities since the mid-2000s, due to increased use of diesel in vehicles (dieselisation).^43^

Notable differences exist between our study and previously cited literature. First, to our knowledge, this is the only study examining the interaction of urban air pollution on lung function, in which measurements have been obtained postbronchodilator. This measurement removes confounding due to undiagnosed or poorly managed asthma. Our study population lives in one of the most deprived and polluted areas in the UK (appendix 1 p 19) and, perhaps most importantly, this study reflects the contemporary urban air quality environment. By contrast, most of the cohorts investigated in the published literature have linked air pollutant exposures to lung function measurement made in the 1990s to the early-to-mid-2000s (Schultz and colleagues [1994–2001],^44^ Gao and colleagues [1996–2003],^45^ Nordling and colleagues [modelled exposures to children's birth year, 1994–97],^46^ Gehring and colleagues [2000–07],^14^ Eenhuizen and colleagues [exposure at birth address 1996–97, related to airway patency at 4 years of age in 2000–01],^47^ Hoek and colleagues [1988–99],^48^ Roy and colleagues [1993–96]^49^). Consequently, these studies largely report on associations with historic air pollution scenarios and do not fully capture the changes that have occurred in many European cities because of the dieselisation of the vehicle fleet, and the introduction of particle traps. The exception to these reports is the follow-up of the Californian Children's Health Study,^19^ but the proportion of diesels within the US vehicle fleet is much lower than that in European cities.^43^ Globally, diesel cars have increased their share of the car market worldwide, with much of this growth in Europe, where more than half of new cars are fuelled by diesel.^43^

Our findings have important implications for the health of children living in central London and other high pollution urban environments, particularly in Europe where diesel vehicles make up a substantial proportion of the vehicle fleet.^43^ We have provided quantitative evidence of improvements of NO_2_ concentrations within London's LEZ, representing a proxy for diesel tailpipe emissions. The extent to which these improvements can be solely attributed to the tightening of emission standards within the zone in early 2011 is difficult to ascertain, given the number of other actions that have been ongoing, which have been summarised previously.^37^ The fact that these benefits have been achieved against the backdrop of the delayed implementation of the LEZ's later phases and vehicles not performing to European emission standards during real-life driving conditions^50^ suggests that further tightening of regulations will achieve more pronounced pollutant reductions. Although longer-term evaluations of the LEZs impact are in progress, the introduction and rigorous evaluation of zones with greater reductions in pollutant concentrations both in London and other cities are clearly warranted.

The impacts on children's FVC might appear small (0·0023 L/μg per m^3^ of NO_2_); however, these decrements need to be viewed against the very high annual exposures in the study areas. Over the full 5 years of the study, the average exposure to NO_2_ equated to a projected FVC loss of between 89·0 mL and 99·6 mL. Given the average FVC seen within the study population of 1·87 L, this result equates to a loss of between 4·8% and 5·3%. These calculations require certain caveats because they imply a simple linear association and the absence of the threshold concentration, but nevertheless they raise great public health concerns. Although changes of this magnitude are unlikely to be clinically significant in the healthy population, the more important issue is whether this change results in an inability to attain maximal lung development in adulthood, with potential impacts on long-term health.^18^ Until longer-term impacts are known, clinicians should consider advising parents of children with lung disease to avoid living in high pollutant areas, or to adopt personal mitigation measures to limit their exposures.

Research is needed to determine whether lung deficits arising from air pollutant exposures in childhood persist into adulthood and to identify which factors lead to increased susceptibility to or protection from these adverse effects. Although long-term data from California provide grounds for cautious optimism that poor lung growth trajectories can be improved by reducing pollution,^19^ this idea must be viewed in the context of the much higher exposure concentrations seen in our study and the different mix of pollutant sources, particularly diesel vehicles. The extent to which observations in the USA are generalisable to the European region is not clear. Our study also only infers changes in lung growth, from the association of FVC with long-term, and not short-term, exposures. Further studies should use longitudinal cohort designs to assess health effects of interventions that will deliver more substantial improvements in urban air quality.
