# Supplementary material for: Impact of London's low emission zone on air quality and children's respiratory health: a sequential annual cross-sectional study
Source: Lancet Public Health. 2018 Nov 15;4(1):e28–40. doi: 10.1016/S2468-2667(18)30202-0 (PMC6323357; doi:10.1016/S2468-2667(18)30202-0)
Supplement: Supplementary appendix 1 [file mmc1.pdf]

THE LANCET
Public Health

Supplementary appendix

This appendix formed part of the original submission and has been peer reviewed. We post it as supplied by the authors.

Supplement to: Mudway IS, Dundas I, Wood HE, et al. Impact of London's low emission zone on air quality and children's respiratory health: a sequential annual cross-sectional study. *Lancet Public Health* 2018; published online Nov 14. http://dx.doi.org/10.1016/S2468-2667(18)30202-0.

Supplementary Materials

Impact of London's Low Emission Zone on air quality and children's respiratory health: a sequential annual cross-sectional study

Ian S. Mudway PhD[1], Isobel Dundas PhD[2], Helen E. Wood DPhil[1], Nadine Marlin MSc[2], Jeenath B. Jamaludin PhD[1,3], Stephen A. Bremner PhD[2], Louise Cross RSN[2], Andrew Grieve MSc[1], Alex Nanzer PhD[2], Ben M. Barratt PhD[1], Sean Beevers PhD[1], David Dajnak PhD[1], Gary W. Fuller PhD[1], Anna Font PhD[1], Aziz Sheikh MD[5], Robert Walton MD[2], Jonathan Grigg MD[2,4], Frank J. Kelly PhD ScD[1], Tak Lee DsC[4,6], Chris J Griffiths DPhil[2,4]

Contents

Materials are presented in the order they are referred to within the main manuscript

4	Background to London's Low Emission Zone.
5	**Table s1**: Elements of the health assessment and indicators obtained.
6	Air pollutant exposure attribution (expanded).
7	**Table s2**: Values of the spearman correlation coefficient, root mean square error, normalised mean gross error and normalised mean bias for observed vs. modelled annual average concentrations for each of the years between 2008 and 2013.
8	Nowcast method description.
8-10	Nowcast method evaluation.
9	**Table s3**: Results of the Nowcast evaluation Jan 2009 to May 2010, representing mean performance across all model runs.
9	**Table s4**: Individual site results of the Nowcast evaluation Jan 2009 to May 2010 for 16 PM_{10} sites (c. 11,000 predictions).
10	**Table s5**: Nowcast evaluation Jan 2009 to May 2010 run in retrospective mode for 12 NO_2 sites (c. 197,000 predictions).
11	**Table s6:** Pearson correlation between 6-9am NOWCAST derived pollutant attributions for participants across each of the 5 study years, plus pilot.
12	**Table s7:** Pearson correlation between 24-hour NOWCAST derived pollutant attributions for participants across each of the 5 study years, plus pilot.
13	**Table s8**: Pearson correlation between 7-day NOWCAST derived pollutant attributions for participants across each of the 5 study years, plus pilot.
14	**Table s9:** Pearson correlation between annual derived pollutant attributions for participants across each of the 5 study years, plus pilot.
15	Lung function assessments (expanded).
16	**Table s10**: Definitions of current and lifetime respiratory/allergic symptoms.
17	**Table s11**: Lung function post-bronchodilator by study year.
18	**Table s12**: Comparison of children included and excluded from primary analysis.
19	**Table s13:** Key characteristics of the study boroughs within central London, with comparators for London and the UK. Data are provided for 2009 (study year 1) and 2014 (study year 5), with ethnic diversity based on 2011 census data and IMD presented reference years 2007, 2010 and 2015.
20	**Table s14**: Effect estimates for the association between demographic variables and measures of Lung Functions (study years 1-5). Associations are adjusted for all annual pollutant exposures, based on residential address.
21	**Figure s1:** Annual NOx maps for the years 2008 -2013.
22	**Figure s2:** Annual PM_{10} maps for the years 2008 -2013.
23	**Figure s3**: Annual $PM_{2.5}$ maps for the years 2008 -2013.
24	**Table s15**: Modelled annual pollutant attributions (Home and Home+School) by study year ($\mu g/m^3$ – median with 25th and 75th percentiles).
25	**Figure s4**: Running annual mean PM_{10} and $PM_{2.5}$ concentrations at inner and outer London roadside and background sites within and surrounding the study area from the beginning of 2006 to 2014 (panels A and D). Air pollution trends are shown relative the three phases of the LEZ. Panels B and C show Forest plots of roadside and background trends in PM_{10} (changes in air pollutant concentrations per year) across the period 2008-2013 by site and aggregated across sites. Panels E and F illustrate the equivalent data for $PM_{2.5}$. *** significance at the <0.001, ** significance at <0.01, * significance at <0.05, per site and aggregated across sites.

26	**Table s16:** Effects of air pollutants on post-bronchodilator FEV_1 in study years 1-5 and with the pilot year included.
27	**Table s17**: Effects of air pollutants on post-bronchodilator FVC in study years 1-5 and with the pilot year included.
28	**Table s18**: Effects of air pollutants on post-bronchodilator FEV_1 and FVC in study years 1-5. Data are expressed as a change in volume (L) per IQR of modelled pollutant concentrations at residential address and weighted for the period spent at school.
29	**Table s19**: Effects of air pollutants on pre-bronchodilator FEV_1 and FVC in study years 1-5.
30	**Figure s5**: Proportion of children with lung function (FEV_1 and FVC) below 90, 85 and 80% of predicted. Contributions of the 4-main ethnic groups are shown.
31	**Figure s6**: Odds ratios and 95% confidence intervals for current and lifetime allergic and respiratory symptoms in relation to annual pollutant exposure attributions based on residential address.
32	**Figure s7**: Yearly prevalence of rhinitis (panel A) and lifetime asthma (panel B) in relation to annual exposure to NOx, NO_2, PM_{10} and $PM_{2.5}$, based on residential address.
33	Supplementary References

Background to London's Low Emission Zone

London's LEZ is roughly contiguous with London's M25 orbital motorway and encompasses approximately 8·5 million residents. Its stated goal was two-fold, to "*move London closer to achieving target air quality objective and limit values*", and "*to improve the health and quality of life of people who work in and visit London, through improving air quality*".[S1]

The scheme was introduced in stages. Phase 1, introduced in February 2008, applied to diesel-powered heavy goods vehicles (HGVs) weighing greater than 12 tonnes. Phase 2 followed in July 2008 applying to HGVs over 3.5 tonnes. Phases 3 and 4 were introduced simultaneously in January 2012, restricting access to heavier Light Goods Vehicles and mini-buses not meeting the Euro III standard, and increasing the restriction on buses, coaches and HGVs greater than 3.5 tonnes from emission standard Euro III to Euro IV. In this paper for simplicity we refer to three phases of introduction, with phase three, encompassing the LEZ phases 3 and 4. For a fuller description of the LEZ we refer the reader to the review of Hollman et al (2015).[S2]

Over a 10-year horizon the predicted impact of the Low Emission Zone within Greater London was to regain 5,000 years of life that would otherwise have been lost, reduce by a quarter of a million the number of restricted activity days, reduce the severity of 300,000 acute respiratory episodes, and deliver health benefits to the value of up to £450m.

Table s1: Elements of the health assessment and indicators obtained

Element	Health indicators obtained
Height, seated height and weight measurement	Height and weight (for calculating predicted values for spirometry, body proportions, BMI)
Spirometry (pre- and post-salbutamol)	Lung function (FEV_1, FVC)
Exhaled nitric oxide	FeNO
Urine sample	ETS exposure, 8-isoprostane and 8-oxodG (markers of systemic inflammation), urinary metals (markers of traffic exposure)
Saliva sample	Cortisol (marker of stress), IgA (marker of innate mucosal immunity)
Saliva swab<br>ISAAC questionnaire	DNA extracted for SNP analysis, telomere length<br>Respiratory and allergic symptoms (wheeze/asthma, rhinitis/hay fever, eczema), ETS exposure, use of paracetamol and ibuprofen, use of gas stove at home
Finger prick blood sample*	Vitamin D level
Induced sputum^	Respiratory macrophages for assessment of macrophage black carbon (marker of traffic exposure)

BMI, body mass index; FEV_1, forced expiratory volume in 1 sec; FVC, forced vital capacity; FeNO, fraction of exhaled nitric oxide; ETS, environmental tobacco smoke; 8-oxodG, 8-hydroxy-2-deoxy Guanosine; IgA, immunoglobulin A; SNP, single nucleotide polymorphism (in selected genes of interest); * added to study protocol for final year only, Year 6; ^ only undertaken during selected visits, with selected children

Air pollutant exposure attribution

Annual NO_2, NOx, PM_{10} and $PM_{2.5}$ concentrations were derived using the KCLurban model[S3] using ADMS dispersion model v4 and road source model v2.3 (CERC19),[S4] measured hourly meteorological data, empirically derived NO-NO_2-O_3 and PM relationships and emissions from the London Atmospheric Emissions Inventory.[S5] Separate annual surface maps for Greater London were produced for the years 2008 to 2013, available at a 20m×20m grid point resolution, linked to the following health assessment periods: Nov 2008 – March 2009 through to Nov 2012 – April 2014. Each yearly model reflected a range of pollutant sources and emissions, including major and minor roads, with detailed information on vehicle stock, traffic flows, and speed on a link-by-link basis. Other sources within the model included large and small regulated industrial processes, boiler plants, domestic and commercial combustion sources, agriculture, rail, ships, airports, and pollution carried into the area by prevailing winds. A comprehensive description of this model has been published previously, together with information on validation against measurement[S6] and its performance against other urban dispersion models.[S7] Further information on the performance of the model against measurements recorded at LAQN monitoring sites has been summarized previously: https://www.kcl.ac.uk/lsm/research/divisions/aes/research/ERG/research-projects/traffic/TRAFFIC-SM-Air-pollution-Model.pdf, but for the purposes of this study we have updated the material in this online resource to include the study years from the present investigation, **Table s2**. All exposures were based on the annual mean within a 20m radius buffer zone around the residential address of the child. Exposure estimates were weighted for periods spent at the home (H) and school (S) address points based on the following criteria: that each child spent 84.4% of their time at home and 15.6% at school, based on a 7-hour school day, for 5 days per week, 39 weeks per year. Thus, each child's weighted exposure was estimated by $E_{H+S} = 0.884*E_H + 0.156*E_S$.

Table s2: Values of the spearman correlation coefficient (r), root mean square error (RMSE), normalised mean gross error (NMGE) and normalised mean bias (NMB) for observed vs. modelled annual average concentrations for each of the years between 2008 and 2013

Year	Pollutant	Number of monitoring sites (n)	Normalised mean bias (NMB)	Normalised mean gross error (NMGE)	Root mean square error (RMSE)	Spearman correlation coefficient (r)
2008	NOx	100	−0.120	0.2	18	0.92
2009	NOx	96	−0.091	0.23	22	0.79
2010	NOx	87	−0.132	0.23	22	0.86
2011	NOx	74	-0.196	0.27	27	0.70
2012	NOx	65	-0.199	0.27	30	0.75
2013	NOx	68	-0.222	0.28	28	0.77
2008	NO_2	100	−0.011	0.13	5.1	0.93
2009	NO_2	96	0.031	0.15	5.5	0.85
2010	NO_2	87	−0.014	0.14	5.8	0.89
2011	NO_2	74	-0.036	0.16	6.6	0.77
2012	NO_2	65	0.003	0.17	6.7	0.81
2013	NO_2	68	-0.001	0.16	6.3	0.82
2008	PM_{10}	76	−0.0067	0.104	4.1	0.78
2009	PM_{10}	74	0.0418	0.117	3.7	0.78
2010	PM_{10}	68	0.0415	0.13	4.2	0.71
2011	PM_{10}	58	0.0636	0.11	3.9	0.79
2012	PM_{10}	49	0.0606	0.12	3.9	0.78
2013	PM_{10}	43	0.0813	0.16	5.1	0.60
2008	$PM_{2.5}$	15	0.0019	0.11	2	0.74
2009	$PM_{2.5}$	21	0.104	0.15	2.8	0.59
2010	$PM_{2.5}$	20	0.0157	0.15	2.8	0.26
2011	$PM_{2.5}$	19	0.0534	0.16	2.8	0.25
2012	$PM_{2.5}$	20	0.0543	0.15	2.6	0.27
2013	$PM_{2.5}$	20	0.1361	0.17	3.1	0.55

Nowcast method description

Time series exposure estimates were derived at the address point by scaling modelled annual mean concentrations according to a 'Nowcast' factor (f) calculated for each pollutant for each day of the analysis period. Similar methods have been employed previously.[S8,S9] The Nowcast factor (f) was defined as the ratio between concentration of each pollutant measured by a local subset of continuous air pollution monitoring sites (L) in the defined prior period (t), and the annual mean (a) measured by the same sites. Thus, the acute exposure concentration estimate [P] at time t for point (x,y) was calculated as:

$$[\mathrm{P}]_{\mathrm{t}}^{(\mathrm{x,y})} = \mathrm{f}.[\mathrm{P}]_{\mathrm{a}}^{(\mathrm{x,y})} \quad \text{where } \mathrm{f} = \frac{[\mathrm{P}]_{\mathrm{t}}^{\mathrm{L}}}{[\mathrm{P}]_{\mathrm{a}}^{\mathrm{L}}}$$

For this study 'Nowcast' scaling factors were calculated for each day of the study period to obtain a spatially resolved time-series of daily mean $PM_{2.5}$, PM_{10}, NO_2 and NO_X concentrations. To derive NOx and NO_2, scaling factors measurements were averaged across 14-17 urban background and roadside sites within and surrounding the London Boroughs of Tower Hamlets and Hackney, based on data availability. For the PM_{10} and $PM_{2.5}$ scaling factors measurements from 9-13 and 14-20 background and roadside sites were averaged, respectively. All measurements were taken from the London Air Quality Monitoring Network (LAQN, www.londonair.org.uk).

Nowcast method evaluation

The Nowcast method has previously been evaluated according to the metrics recommended for the evaluation of air quality models by the Department for Environment, Food & Rural Affairs (DEFRA).[S10] NO_2 and PM_{10} Nowcast factors were calculated for address points coincidental with a randomly selected subset of London Air Quality Monitoring Network (LAQN, www.londonair.org.uk) monitoring sites and compared to measured concentrations over two years from June 2008 to May 2010. The test included 268,000 comparisons for PM_{10} and 197,000 for NO_2. The results of the Nowcast evaluation are shown in **Table s3**. There was a bias evident in both pollutant models, with the Nowcast tending to under predict measured concentrations. This is due to two factors: (1) the 20 m resolution of the model means that it cannot reflect high concentrations within a few metres of roads and may underestimate kerbside concentrations and (2) part of the Nowcast calculation is based on median rather than mean concentrations to avoid undue influence from extraneous high or low measurements. The normalised mean bias for NO_2 and PM_{10} is

within the DEFRA recommended bounds of ± 20% and the FAC2 (fraction of predictions within a factor of 0.5 to 2) is well above the recommended 50%.

Table s3: Results of the Nowcast evaluation Jan 2009 to May 2010, representing mean performance across all model runs.

	Measured Mean $\mu g\ m^{-3}$	Modelled Mean $\mu g\ m^{-3}$	Mean error $\mu g\ m^{-3}$	Standard deviation $\mu g\ m^{-3}$	Normalised mean bias %	Normalised mean gross error %	FAC2 %
**PM_{10}**	25	23	2	7	9	19	99
**NO_2**	56	52	5	30	8	34	85

Table s4 shows Nowcast performance at each of the 16 PM_{10} monitoring sites in the evaluation. The FAC2 is greater than 50% at all sites meeting the DEFRA criteria. The normalised mean bias is inside the DEFRA recommended range of ± 20% at 13 sites. At three sites close to roads (highlighted in grey) normalised mean bias is outside the range of ± 20%. At these sites the 20 m model resolution cannot fully represent peak concentrations within a few metres of traffic sources.

Table s4: Individual site results of the Nowcast evaluation Jan 2009 to May 2010 for 16 PM_{10} sites (c. 11,000 predictions). Letters following the site code denote the site type, (K) = kerbside, (R) = roadside and (B) = background or suburban.

Site	Measured Mean $\mu g\ m^{-3}$	Modelled Mean $\mu g\ m^{-3}$	Mean error $\mu g\ m^{-3}$	Standard deviation $\mu g\ m^{-3}$	Normalised mean bias %	Normalised mean gross error %	FAC2 %
**BL0 (B)**	19	19	0	4	1	15	100
**BN1(K)**	23	25	-2	3	-8	11	100
**BT1(B)**	18	17	0	3	2	9	100
**CD3(R)**	30	26	4	5	13	16	100
**GR4(B)**	24	19	5	4	19	20	100
**HG1(R)**	22	19	3	4	14	16	99
**HR1(B)**	17	17	0	2	-2	9	100
**HS5(R)**	32	21	11	15	34	35	89
**IS2(R)**	27	25	3	4	10	15	100
**KC1(B)**	21	19	2	2	10	11	100
**KC2(R)**	28	26	1	4	4	12	100
**LH2(B)**	23	20	3	4	14	16	100
**MY1(K)**	36	44	-8	13	-21	33	96
**ST4(K)**	25	17	8	4	31	31	97
**TH1(B)**	22	19	3	3	13	14	100
**TH4(R)**	34	29	5	7	14	18	99

Notes: Bloomsbury, Russell Square background (BL0), Barnet, Tally Ho Corner kerbside (BN1), Brent, Kingsbury background (BT1), Camden, Shaftesbury Avenue roadside (CD3); Greenwich, Eltham background (GR4); Haringey, Town Hall background (HG1); Harrow, Stanmore background (HR1); Hounslow, Brentford roadside (HS5); Islington, Holloway Road (IS2); Kensington & Chelsea, North Kensington background (KC1); Kensington & Chelsea, Cromwell Road (KC2); London Heathrow background (LH2); Marylebone Road (MY1); Sutton, Wallington roadside (ST4); Tower Hamlets, Popular background (TH1); Tower Hamlets, Blackwall Tunnel roadside (TH4).

Table s5 shows the Nowcast performance at 12 NO_2 sites. The FAC2 is greater than 50% at all sites meeting the Defra criteria. The normalised mean bias is inside the recommended range of ± 20% at nine of the twelve sites. It was outside the range of ± 20% at two sites close to roads (highlighted in grey) where the 20 m model resolution cannot fully represent peak concentrations within a few metres of traffic sources. The normalised mean bias was also outside ± 20% at one background site.

Table s5: Nowcast evaluation Jan 2009 to May 2010 run in retrospective mode for 12 NO_2 sites (c. 197,000 predictions). Letters following the site code denote the site type, (K) = kerbside, (R) = roadside and (B) = background or suburban.

Site	Measured Mean µg m^{-3}	Modelled Mean µg m^{-3}	Mean error µg m^{-3}	Standard deviation µg m^{-3}	Normalised mean bias %	Normalised mean gross error %	FAC2 %
**BN1(K)**	68	61	7	24	11	29	91
**BT1(B)**	32	31	1	12	3	30	86
**CD3(R)**	84	65	18	25	22	30	86
**EA1(B)**	40	41	-1	11	-3	21	97
**EA2(R)**	58	53	5	22	9	26	94
**GR4(B)**	24	30	-6	10	-23	38	73
**HR1(B)**	26	29	-3	11	-10	34	82
**HS5(R)**	59	48	11	19	19	28	90
**IS2(R)**	59	62	-2	20	-4	28	92
**MY1(K)**	107	116	-9	62	-9	48	72
**ST4(K)**	76	42	34	42	44	50	63
**TH1(B)**	37	37	1	10	2	19	99

Notes: Barnet, Tally Ho Corner kerbside (BN1), Brent, Kingsbury background (BT1), Camden, Shaftesbury Avenue roadside (CD3); Ealing, Ealing Town Hall background (EA1); Ealing, Acton Town Hall roadside (EA2); Greenwich, Eltham background (GR4); Harrow, Stanmore background (HR1); Hounslow, Brentford roadside (HS5); Islington, Holloway Road (IS2); Marylebone Road (MY1); Sutton, Wallington roadside (ST4); Tower Hamlets, Popular background (TH1).

Table s6: Pearson correlation between 6-9am NOWCAST derived pollutant attributions for participants across each of the 5 study years, plus pilot.

Pilot		NOx	NO_2	PM_{10}	$PM_{2.5}$	NOx	NO_2	PM_{10}	$PM_{2.5}$
		Home				Home+school			
NOx	Home	1.00							
NO_2		0.96	1.00						
PM_{10}		0.85	0.79	1.00					
$PM_{2.5}$		0.78	0.69	0.96	1.00				
NOx	Home+school	1.00	0.96	0.85	0.78	1.00			
NO_2		0.95	1.00	0.79	0.69	0.96	1.00		
PM_{10}		0.85	0.79	1.00	0.97	0.85	0.79	1.00	
$PM_{2.5}$		0.77	0.68	0.96	1.00	0.78	0.69	0.96	1.00

**Year 1**		NOx	NO_2	PM_{10}	$PM_{2.5}$	NOx	NO_2	PM_{10}	$PM_{2.5}$
		Home				Home+school			
NOx	Home	1.00							
NO_2		0.96	1.00						
PM_{10}		0.86	0.77	1.00					
$PM_{2.5}$		0.86	0.79	0.98	1.00				
NOx	Home+school	1.00	0.96	0.87	0.87	1.00			
NO_2		0.96	1.00	0.78	0.80	0.96	1.00		
PM_{10}		0.85	0.77	1.00	0.98	0.86	0.78	1.00	
$PM_{2.5}$		0.86	0.78	0.98	1.00	0.87	0.79	0.98	1.00

**Year 2**		NOx	NO_2	PM_{10}	$PM_{2.5}$	NOx	NO_2	PM_{10}	$PM_{2.5}$
		Home				Home+school			
NOx	Home	1.00							
NO_2		0.93	1.00						
PM_{10}		0.59	0.67	1.00					
$PM_{2.5}$		0.67	0.71	0.96	1.00				
NOx	Home+school	1.00	0.93	0.60	0.69	1.00			
NO_2		0.92	1.00	0.69	0.73	0.93	1.00		
PM_{10}		0.58	0.66	1.00	0.96	0.59	0.68	1.00	
$PM_{2.5}$		0.67	0.70	0.96	1.00	0.68	0.72	0.96	1.00

**Year 3**		NOx	NO_2	PM_{10}	$PM_{2.5}$	NOx	NO_2	PM_{10}	$PM_{2.5}$
		Home				Home+school			
NOx	Home	1.00							
NO_2		0.89	1.00						
PM_{10}		0.63	0.58	1.00					
$PM_{2.5}$		0.51	0.39	0.94	1.00				
NOx	Home+school	1.00	0.89	0.63	0.52	1.00			
NO_2		0.89	1.00	0.59	0.39	0.89	1.00		
PM_{10}		0.63	0.58	1.00	0.94	0.63	0.59	1.00	
$PM_{2.5}$		0.51	0.38	0.94	1.00	0.52	0.39	0.94	1.00

**Year 4**		NOx	NO_2	PM_{10}	$PM_{2.5}$	NOx	NO_2	PM_{10}	$PM_{2.5}$
		Home				Home+school			
NOx	Home	1.00							
NO_2		0.93	1.00						
PM_{10}		0.59	0.55	1.00					
$PM_{2.5}$		0.60	0.57	0.97	1.00				
NOx	Home+school	1.00	0.93	0.59	0.60	1.00			
NO_2		0.93	1.00	0.55	0.57	0.93	1.00		
PM_{10}		0.59	0.55	1.00	0.97	0.59	0.55	1.00	
$PM_{2.5}$		0.60	0.57	0.97	1.00	0.60	0.57	0.97	1.00

**Year 5**		NOx	NO_2	PM_{10}	$PM_{2.5}$	NOx	NO_2	PM_{10}	$PM_{2.5}$
		Home				Home+school			
NOx	Home	1.00							
NO_2		0.91	1.00						
PM_{10}		0.62	0.52	1.00					
$PM_{2.5}$		0.73	0.69	0.85	1.00				
NOx	Home+school	1.00	0.91	0.62	0.73	1.00			
NO_2		0.90	1.00	0.52	0.69	0.90	1.00		
PM_{10}		0.61	0.51	1.00	0.85	0.61	0.51	1.00	
$PM_{2.5}$		0.73	0.69	0.85	1.00	0.73	0.68	0.85	1.00

Table s7: Pearson correlation between 24-hour NOWCAST derived pollutant attributions for participants across each of the 5 study years, plus pilot.

**Pilot**		NOx	NO_2	PM_{10}	$PM_{2.5}$	NOx	NO_2	PM_{10}	$PM_{2.5}$
		Home				Home+school			
NOx	Home	1.00							
NO_2		0.94	1.00						
PM_{10}		0.95	0.88	1.00					
$PM_{2.5}$		0.90	0.78	0.94	1.00				
NOx	Home+school	1.00	0.94	0.95	0.90	1.00			
NO_2		0.94	1.00	0.88	0.78	0.94	1.00		
PM_{10}		0.95	0.88	1.00	0.94	0.95	0.88	1.00	
$PM_{2.5}$		0.89	0.77	0.94	1.00	0.90	0.78	0.94	1.00

**Year 1**		NOx	NO_2	PM_{10}	$PM_{2.5}$	NOx	NO_2	PM_{10}	$PM_{2.5}$
		Home				Home+school			
NOx	Home	1.00							
NO_2		0.97	1.00						
PM_{10}		0.76	0.74	1.00					
$PM_{2.5}$		0.69	0.66	0.93	1.00				
NOx	Home+school	1.00	0.97	0.77	0.71	1.00			
NO_2		0.97	1.00	0.75	0.68	0.97	1.00		
PM_{10}		0.75	0.73	1.00	0.93	0.76	0.74	1.00	
$PM_{2.5}$		0.69	0.66	0.93	1.00	0.71	0.67	0.93	1.00

**Year 2**		NOx	NO_2	PM_{10}	$PM_{2.5}$	NOx	NO_2	PM_{10}	$PM_{2.5}$
		Home				Home+school			
NOx	Home	1.00							
NO_2		0.87	1.00						
PM_{10}		0.83	0.81	1.00					
$PM_{2.5}$		0.87	0.80	0.96	1.00				
NOx	Home+school	1.00	0.87	0.84	0.88	1.00			
NO_2		0.87	1.00	0.82	0.81	0.87	1.00		
PM_{10}		0.82	0.81	1.00	0.96	0.83	0.82	1.00	
$PM_{2.5}$		0.87	0.80	0.96	1.00	0.88	0.81	0.96	1.00

**Year 3**		NOx	NO_2	PM_{10}	$PM_{2.5}$	NOx	NO_2	PM_{10}	$PM_{2.5}$
		Home				Home+school			
NOx	Home	1.00							
NO_2		0.89	1.00						
PM_{10}		0.70	0.59	1.00					
$PM_{2.5}$		0.65	0.50	0.98	1.00				
NOx	Home+school	1.00	0.89	0.71	0.66	1.00			
NO_2		0.89	1.00	0.60	0.51	0.89	1.00		
PM_{10}		0.69	0.59	1.00	0.98	0.70	0.60	1.00	
$PM_{2.5}$		0.65	0.50	0.98	1.00	0.66	0.51	0.98	1.00

**Year 4**		NOx	NO_2	PM_{10}	$PM_{2.5}$	NOx	NO_2	PM_{10}	$PM_{2.5}$
		Home				Home+school			
NOx	Home	1.00							
NO_2		0.94	1.00						
PM_{10}		0.48	0.51	1.00					
$PM_{2.5}$		0.63	0.64	0.94	1.00				
NOx	Home+school	1.00	0.94	0.48	0.63	1.00			
NO_2		0.94	1.00	0.51	0.64	0.94	1.00		
PM_{10}		0.48	0.51	1.00	0.94	0.48	0.51	1.00	
$PM_{2.5}$		0.63	0.64	0.94	1.00	0.63	0.64	0.94	1.00

**Year 5**		NOx	NO_2	PM_{10}	$PM_{2.5}$	NOx	NO_2	PM_{10}	$PM_{2.5}$
		Home				Home+school			
NOx	Home	1.00							
NO_2		0.95	1.00						
PM_{10}		0.80	0.70	1.00					
$PM_{2.5}$		0.85	0.78	0.83	1.00				
NOx	Home+school	1.00	0.95	0.81	0.86	1.00			
NO_2		0.95	1.00	0.71	0.79	0.95	1.00		
PM_{10}		0.79	0.69	1.00	0.83	0.80	0.70	1.00	
$PM_{2.5}$		0.85	0.78	0.84	1.00	0.86	0.78	0.83	1.00

Table s8: Pearson correlation between 7-day NOWCAST derived pollutant attributions for participants across each of the 5 study years, plus pilot.

Pilot		NOx	NO_2	PM_{10}	$PM_{2.5}$	NOx	NO_2	PM_{10}	$PM_{2.5}$
		Home				Home+school			
NOx	Home	1.00							
NO_2	Home	0.97	1.00						
PM_{10}	Home	0.79	0.69	1.00					
$PM_{2.5}$	Home	0.80	0.70	0.98	1.00				
NOx	Home+school	1.00	0.97	0.81	0.82	1.00			
NO_2	Home+school	0.97	1.00	0.70	0.72	0.97	1.00		
PM_{10}	Home+school	0.78	0.68	1.00	0.98	0.79	0.69	1.00	
$PM_{2.5}$	Home+school	0.79	0.69	0.98	1.00	0.81	0.70	0.98	1.00

Year 1		NOx	NO_2	PM_{10}	$PM_{2.5}$	NOx	NO_2	PM_{10}	$PM_{2.5}$
		Home				Home+school			
NOx	Home	1.00							
NO_2	Home	0.97	1.00						
PM_{10}	Home	0.79	0.74	1.00					
$PM_{2.5}$	Home	0.74	0.67	0.95	1.00				
NOx	Home+school	1.00	0.97	0.80	0.76	1.00			
NO_2	Home+school	0.97	1.00	0.75	0.69	0.97	1.00		
PM_{10}	Home+school	0.78	0.73	1.00	0.95	0.80	0.75	1.00	
$PM_{2.5}$	Home+school	0.73	0.67	0.95	1.00	0.76	0.69	0.95	1.00

Year 2		NOx	NO_2	PM_{10}	$PM_{2.5}$	NOx	NO_2	PM_{10}	$PM_{2.5}$
		Home				Home+school			
NOx	Home	1.00							
NO_2	Home	0.92	1.00						
PM_{10}	Home	0.31	0.28	1.00					
$PM_{2.5}$	Home	0.38	0.30	0.94	1.00				
NOx	Home+school	0.99	0.91	0.30	0.38	1.00			
NO_2	Home+school	0.90	0.99	0.28	0.29	0.91	1.00		
PM_{10}	Home+school	0.29	0.26	1.00	0.94	0.29	0.26	1.00	
$PM_{2.5}$	Home+school	0.37	0.29	0.94	1.00	0.37	0.28	0.94	1.00

Year 3		NOx	NO_2	PM_{10}	$PM_{2.5}$	NOx	NO_2	PM_{10}	$PM_{2.5}$
		Home				Home+school			
NOx	Home	1.00							
NO_2	Home	0.90	1.00						
PM_{10}	Home	0.69	0.73	1.00					
$PM_{2.5}$	Home	0.63	0.61	0.98	1.00				
NOx	Home+school	1.00	0.89	0.69	0.63	1.00			
NO_2	Home+school	0.90	1.00	0.73	0.62	0.90	1.00		
PM_{10}	Home+school	0.69	0.72	1.00	0.98	0.69	0.73	1.00	
$PM_{2.5}$	Home+school	0.63	0.61	0.98	1.00	0.63	0.61	0.98	1.00

Year 4		NOx	NO_2	PM_{10}	$PM_{2.5}$	NOx	NO_2	PM_{10}	$PM_{2.5}$
		Home				Home+school			
NOx	Home	1.00							
NO_2	Home	0.95	1.00						
PM_{10}	Home	0.52	0.54	1.00					
$PM_{2.5}$	Home	0.61	0.61	0.98	1.00				
NOx	Home+school	1.00	0.95	0.51	0.61	1.00			
NO_2	Home+school	0.96	1.00	0.54	0.62	0.95	1.00		
PM_{10}	Home+school	0.51	0.54	1.00	0.98	0.51	0.54	1.00	
$PM_{2.5}$	Home+school	0.61	0.61	0.97	1.00	0.61	0.62	0.98	1.00

Year 5		NOx	NO_2	PM_{10}	$PM_{2.5}$	NOx	NO_2	PM_{10}	$PM_{2.5}$
		Home				Home+school			
NOx	Home	1.00							
NO_2	Home	0.91	1.00						
PM_{10}	Home	0.76	0.76	1.00					
$PM_{2.5}$	Home	0.70	0.68	0.94	1.00				
NOx	Home+school	1.00	0.89	0.78	0.72	1.00			
NO_2	Home+school	0.92	1.00	0.79	0.71	0.91	1.00		
PM_{10}	Home+school	0.76	0.75	1.00	0.94	0.78	0.78	1.00	
$PM_{2.5}$	Home+school	0.70	0.67	0.94	1.00	0.72	0.70	0.94	1.00

Table s9: Pearson correlation between annual derived pollutant attributions for participants across each of the 5 study years, plus pilot.

**Pilot**		NOx	NO_2	PM_{10}	$PM_{2.5}$	NOx	NO_2	PM_{10}	$PM_{2.5}$
		Home				Home+school			
NOx	Home	1.00							
NO_2		1.00	1.00						
PM_{10}		0.97	0.97	1.00					
$PM_{2.5}$		0.97	0.97	0.99	1.00				
NOx	Home+school	1.00	0.99	0.96	0.97	1.00			
NO_2		0.99	1.00	0.95	0.96	1.00	1.00		
PM_{10}		0.97	0.97	1.00	0.99	0.97	0.96	1.00	
$PM_{2.5}$		0.97	0.96	0.98	1.00	0.97	0.97	0.99	1.00

**Year 1**		NOx	NO_2	PM_{10}	$PM_{2.5}$	NOx	NO_2	PM_{10}	$PM_{2.5}$
		Home				Home+school			
NOx	Home	1.00							
NO_2		1.00	1.00						
PM_{10}		0.93	0.94	1.00					
$PM_{2.5}$		0.93	0.94	0.98	1.00				
NOx	Home+school	0.99	0.99	0.91	0.92	1.00			
NO_2		0.99	0.99	0.92	0.93	1.00	1.00		
PM_{10}		0.93	0.94	1.00	0.99	0.93	0.94	1.00	
$PM_{2.5}$		0.92	0.93	0.97	0.99	0.93	0.94	0.98	1.00

**Year 2**		NOx	NO_2	PM_{10}	$PM_{2.5}$	NOx	NO_2	PM_{10}	$PM_{2.5}$
		Home				Home+school			
NOx	Home	1.00							
NO_2		1.00	1.00						
PM_{10}		0.94	0.95	1.00					
$PM_{2.5}$		0.90	0.92	0.99	1.00				
NOx	Home+school	0.98	0.98	0.90	0.87	1.00			
NO_2		0.97	0.98	0.91	0.88	1.00	1.00		
PM_{10}		0.93	0.95	0.98	0.97	0.94	0.95	1.00	
$PM_{2.5}$		0.90	0.91	0.97	0.98	0.90	0.91	0.99	1.00

**Year 3**		NOx	NO_2	PM_{10}	$PM_{2.5}$	NOx	NO_2	PM_{10}	$PM_{2.5}$
		Home				Home+school			
NOx	Home	1.00							
NO_2		1.00	1.00						
PM_{10}		0.94	0.96	1.00					
$PM_{2.5}$		0.92	0.94	0.98	1.00				
NOx	Home+school	0.99	0.98	0.92	0.91	1.00			
NO_2		0.98	0.99	0.93	0.93	1.00	1.00		
PM_{10}		0.94	0.95	0.99	0.98	0.94	0.96	1.00	
$PM_{2.5}$		0.90	0.92	0.95	0.99	0.93	0.94	0.98	1.00

**Year 4**		NOx	NO_2	PM_{10}	$PM_{2.5}$	NOx	NO_2	PM_{10}	$PM_{2.5}$
		Home				Home+school			
NOx	Home	1.00							
NO_2		0.99	1.00						
PM_{10}		0.93	0.96	1.00					
$PM_{2.5}$		0.89	0.92	0.96	1.00				
NOx	Home+school	0.99	0.99	0.92	0.89	1.00			
NO_2		0.99	0.99	0.95	0.92	0.99	1.00		
PM_{10}		0.93	0.95	0.99	0.96	0.93	0.96	1.00	
$PM_{2.5}$		0.87	0.90	0.93	0.99	0.88	0.92	0.96	1.00

**Year 5**		NOx	NO_2	PM_{10}	$PM_{2.5}$	NOx	NO_2	PM_{10}	$PM_{2.5}$
		Home				Home+school			
NOx	Home	1.00							
NO_2		1.00	1.00						
PM_{10}		0.94	0.96	1.00					
$PM_{2.5}$		0.92	0.94	0.97	1.00				
NOx	Home+school	0.99	0.99	0.93	0.92	1.00			
NO_2		0.99	0.99	0.95	0.94	1.00	1.00		
PM_{10}		0.94	0.96	0.99	0.98	0.94	0.96	1.00	
$PM_{2.5}$		0.91	0.93	0.95	0.99	0.93	0.95	0.97	1.00

Lung function assessments

Children's respiratory function was assessed by spirometry (Microlab, Micromedical, Carefusion), performed by trained investigators according to ATS-ERS guidelines[S11] with baseline and post-bronchodilator measurements, following salbutamol, 400 µg, administered by large volume spacer. Before each measurement volume calibration with a 3L syringe was undertaken. A sterile disposable filter/ mouthpiece was attached to the spirometer for each child and the equipment wiped with alcohol wipes between subjects. A maximum of 10 attempts was normally made (exceptionally, more if needed) until three acceptable and two repeatable attempts were attained. Each spirometry measurement aimed to obtain three acceptable and two repeatable attempts both pre and post bronchodilator. A short acting bronchodilator (salbutamol) was administered after baseline spirometry. Four 100 microgram actuations were given from a metered dose inhaler (MDI) through a spacer device (Volumatic). The children took 4 tidal breaths through the Volumatic spacer after each actuation. A minimum of 15 minutes later post bronchodilator spirometry took place.

Quality control was based on the ATS/ERS guidelines[S11], modified for children[S12]. Additional acceptability requirements were: rapid onset of expiration, high well-defined peak flow and a clear plateau on volume-time curve together with no evidence of cough, glottis closure or leak during the manoeuvre (from Asthma UK/ Growing Lungs Guidelines). For quality control and reporting purposes spirometry results were uploaded from the 3 study spirometers to a Carefusion program "Spirometry" where individual inspection of efforts was undertaken in detail. Reporting of results was according to ATS-ERS Guidelines and the best overall individual effort from each child both pre and post salbutamol was selected as the highest value of FEV_1 and FVC, together with FEF_{25-75} from the best combined effort, reported from technically acceptable data, which met reproducibility requirements of 0.15 L agreement for FEV_1 and FVC for 2 efforts. Data were then extracted into Excel and individual efforts were cross checked with original data before being exported into the main study Access database. Raw data were transformed into Z (or standard deviation) scores using most the "All-Age" equations by Stanojevic et al., available for white subjects aged 3 to 80 years of age.[S13]

Table s10: Definitions of current and lifetime respiratory/allergic symptoms

Symptom	Definition
**Current**	
Wheeze	Has your child had wheezing or whistling in the chest in the past 12 months?
	Has your child had wheezing or whistling in the chest in the last 12 months? AND AT LEAST ONE OF: ≥4 attacks of wheezing in the past 12 months.
Severe wheeze	≥1night/week of sleep disturbed by wheezing in the past 12 months; wheezing severe enough to limit speech to only one or two words at a time between breaths in the past 12 months.
Rhinitis	In the past 12 months, has your child had a problem with sneezing, or a runny, or a blocked nose when he/she DID NOT have a cold or the flu?
Eczema	Has your child ever had an itchy rash which was coming and going for at least 6 months? AND Has your child had this itchy rash at any time in the last 12 months? AND Has this itchy rash at any time affected any of the following places: the folds of the elbows, behind the knees, in front of the ankles, under the buttocks, or around the neck, ears or eyes?
**Lifetime**	
Asthma	Has your child ever had asthma?
Hay fever	Has your child ever had hay fever?
Eczema	Has your child ever had eczema?

Table s11: Lung function post-bronchodilator by study year

Year	FEV_1 (L)	FEV_1 (%pred)	FVC (L)	FVC (%pred)	BDR (%)	PEFmax (L/min)
	Mean (SD)					
Pilot (08/09)	1.71 (0.28) N=150	96 (11) N=150	1.92 (0.34) N=148	94 (12) N=148	3.84 (6.69) N=146	216 (46) N=152
1 (09/10)	1.68 (0.28) N=401	92 (12) N=400	1.86 (0.32) N=383	89 (12) N=382	3.96 (6.03) N=394	219 (47) N=398
2 (10/11)	1.70 (0.28) N=399	96 (12) N=399	1.91 (0.31) N=388	95 (11) N=388	4.34 (6.83) N=396	236 (42) N=399
3 (11/12)	1.70 (0.29) N=399	95 (13) N=399	1.91 (0.34) N=395	94 (13) N=395	3.95 (6.50) N=395	229 (42) N=412
4 (12/13)	1.69 (0.27) N=397	93 (11) N=397	1.87 (0.32) N=386	89 (11) N=386	4.24 (6.63) N=388	239 (43) N=397
5 (13/14)	1.67 (0.28) N=417	94 (12) N=417	1.83 (0.32) N=417	91 (12) N=417	3.92 (7.53) N=417	240 (45) N=403
1-5 (09/14)	1.69 (0.28) N=2013	94 (12) N=2012	1.87 (0.32) N=1969	92(12) N=1968	4.08 (6.73) N=1990	233 (44) N=2009

Note: Peak expiratory flow (PEF); bronchodilator response (BDR).

Table s12: Comparison of children included and excluded from primary analysis

Characteristics	FEV_1 or AP exposure missing		FEV_1 and AP exposure recorded	
**Age (years)**	183	8.8(0.3)	1981	8.9(0.3)
**Gender**				
- Male	70	38.3%	943	47.6%
- Female	108	59.0%	1038	52.4%
- Not reported	5	2.7%	0	0.0%
**Reported ethnicity**				
- Asian	73	39.9%	747	37.7%
- Black	51	27.9%	464	23.4%
- White	39	21.3%	489	24.7%
- Mixed / other	17	9.3%	276	13.9%
- Not reported	3	1.6%	5	0.3%
**Height (cm)**	110	133.6 (6.7)	1981	134.0 (6.8)
**Weight (kg)**	110	31.1 (6.7)	1979	32.6 (8.0)
**BMI (kg/m^2)**	110	17.3 (3.0)	1979	18.0 (3.3)
**Deprivation score**	183	44.9 (12.4)	1957	45.0 (11.3)
**Annual mean NOx home ($\mu g/m^3$)**	146	70.6 (10.9)	1981	70.6 (13.3)
**Annual mean NOx school ($\mu g/m^3$)**	147	71.5 (12.9)	1981	71.0 (13.9)
**Annual mean NO_2 home ($\mu g/m^3$)**	146	41.6 (4.3)	1981	41.6 (5.1)
**Annual mean NO_2 school ($\mu g/m^3$)**	147	41.9 (5.1)	1981	41.8 (5.5)
**Annual mean PM_{10} home ($\mu g/m^3$)**	146	24.6 (1.6)	1981	24.6 (1.6)
**Annual mean PM_{10} school ($\mu g/m^3$)**	147	24.6 (1.4)	1981	24.6 (1.5)
**Annual mean $PM_{2.5}$ home ($\mu g/m^3$)**	146	14.8 (1.0)	1981	14.8 (1.1)
**Annual mean $PM_{2.5}$ school ($\mu g/m^3$)**	147	14.8 (1.0)	1981	14.8 (1.1)

Table s13: Key characteristics of the study boroughs within central London, with comparators for London and the UK. Data are provided for 2009 (study year 1) and 2014 (study year 5), with ethnic diversity based on 2011 census data and IMD presented reference years 2007, 2010 and 2015.

**Borough**	**Year**	[1]**Population**	[1]**Male** (%)	[1]**Primary school aged children** (5-11 years)	[2]**Area** (Hectares)	**Pop. density** (people/hectare)	[3]**Ethnicity White, Asian, Black, Mixed/Other** (2011)	[4]**Origin of 3 largest migrant pops.** (2011)	[5,6,7]**IMD** (2007; 2010; 2015)
City of London	2009	7,472	55.7	250	315	23.7	**W** (78.5%); **A** (12.7%); **B** (2.6%); **M/O** (6.0%)	USA France Australia	[8]253, 259, 231
	2014	8,072	55.8	365		25.6			
Greenwich	2009	243,672	49.4	21,019	5,044	48.3	**W** (62.5%); **A** (11.7%); **B** (19.1%); **M/O** (6.7%)	Nigeria Nepal India	17, 19, 78
	2014	268,678	50.1	25,094		53.3			
Hackney	2009	236,622	49.5	20,411	1,905	124.2	**W** (54.7%); **A** (10.5%); **B** (23.1%); **M/O** (11.7%)	Turkey Nigeria Jamaica	1, 1, 11
	2014	263,150	49.7	22,878		138.1			
Tower Hamlets	2009	240,495	51.4	19,900	2,158	111.4	**W** (45.2%); **A** (41.2%); **B** (7.3%); **M/O** (6.4%)	Bangladesh India China	3, 3, 10
	2014	284,015	51.7	24,160		131.6			
Greater London	2009	7,942,594	49.3	644,254	159,471	49.8	**W** (59.8%); **A** (18.4%); **B** (13.3%); **M/O** (8.4%)	India Poland Ireland	-
	2014	8,538,689	49.6	738,729		53.5			
United Kingdom	2009	62,260,486	49.0	4,951,323	24,250,877	2.6	**W** (87.1%); **A** (6.9%); **B** (3.0%); **M/O** (2.9%)	India Poland Pakistan	-
	2014	64,596,752	49.2	5,270,510		2.7			

1 - Source: ONS Mid-year Estimates, http://data.london.gov.uk/dataset/ons-mid-year-population-estimates-custom-age-tables. 2 - Source: GLA (Datastore), http://data.london.gov.uk/dataset/land-area-and-population-density-ward-and-borough. 3 - Source: ONS 2011 Census: Key Statistics for local authorities in England and Wales, https://www.ons.gov.uk/file?uri=/.../r21ewrttableks201ewladv1_tcm77-290595.xls. 4 - Source: Department for Work and Pensions, Foreign nationals who have registered for a national insurance number, http://data.london.gov.uk/dataset/national-insurance-number-registrations-overseas-nationals-borough. 5: The National Archive, http://webarchive.nationalarchives.gov.uk/+/www.communities.gov.uk/communities/neighbourhoodrenewal/deprivation/deprivation07/. 6: Department for Communities and Local Government, English indices of deprivation 2010, https://www.gov.uk/government/statistics/english-indices-of-deprivation-2010. 7: Department for Communities and Local Government, English indices of deprivation 2015, www.gov.uk/government/statistics/english-indices-of-deprivation-2015. 8 - Ranked out of 326 LAs in England (1=most deprived)

Table s14: Effect estimates for the association between demographic variables and measures of Lung Functions (study years 1-5). Associations are adjusted for all annual pollutant exposures, based on residential address.

	FEV_1 (L)		FVC (L)	
	N=1859		N=1859	
	Coeff, 95% CI	p	Coeff, 95% CI	p
**Age**	0.0331 [0.0052,0.0610]	0.02	0.0502 [0.0180,0.0823]	0.002
**Gender**	-0.0859 [-0.1032,-0.0686]	<0.001	-0.1383 [-0.1582,-0.1184]	<0.001
**Height**	0.0266 [0.0251,0.0281]	<0.001	0.0284 [0.0266,0.0301]	<0.001
**BMI**	0.0065 [0.0037,0.0093]	<0.001	0.0138 [0.0106,0.0171]	<0.001
**IMD score**	-0.0005 [-0.0013,0.0003]	0.25	-0.0004 [-0.0013,0.0005]	0.38
**ETS CCR**	-0.0144 [-0.0387,0.0098]	0.24	-0.0155 [-0.0434,0.0124]	0.28
**Ethnicity Black vs Asian**	-0.1182 [-0.1431,-0.0932]	<0.001	-0.1103 [-0.1388,-0.0819]	<0.001
**Ethnicity White vs Asian**	0.1217 [0.0974,0.1460]	<0.001	0.1644 [0.1368,0.1920]	<0.001
**Ethnicity Mixed vs Asian**	0.1063 [0.0784,0.1342]	<0.001	0.1281 [0.0960,0.1601]	<0.001
**Study year 2 vs 1**	0.0238 [-0.0491,0.0967]	0.52	0.0669 [-0.0225,0.1562]	0.14
**Study year 3 vs 1**	0.0259 [-0.0780,0.1298]	0.63	0.0378 [-0.0922,0.1677]	0.57
**Study year 4 vs 1**	0.0072 [-0.0391,0.0536]	0.76	0.0059 [-0.0513,0.0631]	0.84
**Study year 5 vs 1**	-0.0051 [-0.1164,0.1061]	0.93	-0.0342 [-0.1729,0.1046]	0.63

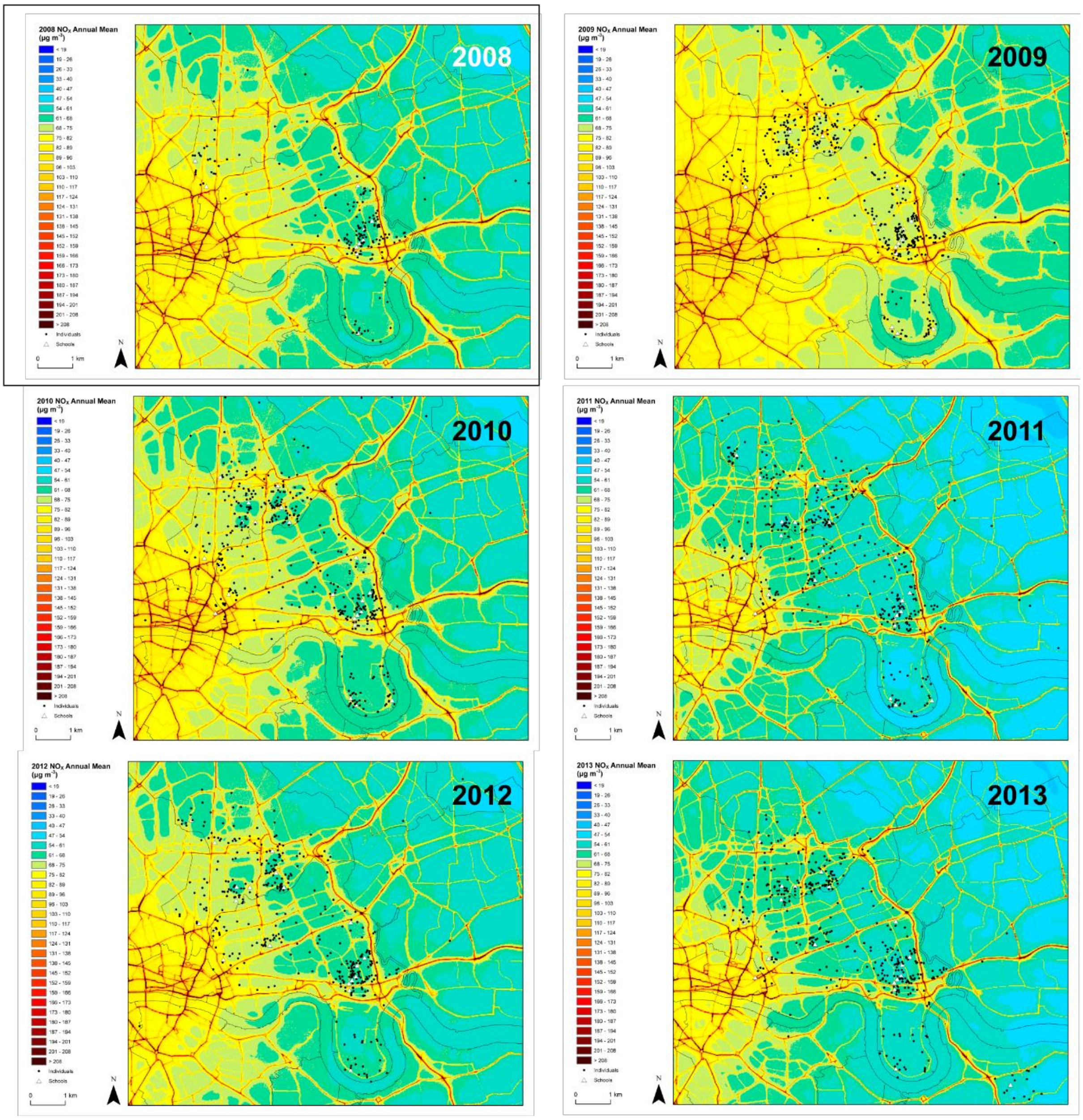

Figure s1: Annual NOx maps for the years 2008 -2013. Individual points reflect the residential address of the volunteers tested during the winter periods, with the school locations highlighted using open triangles.

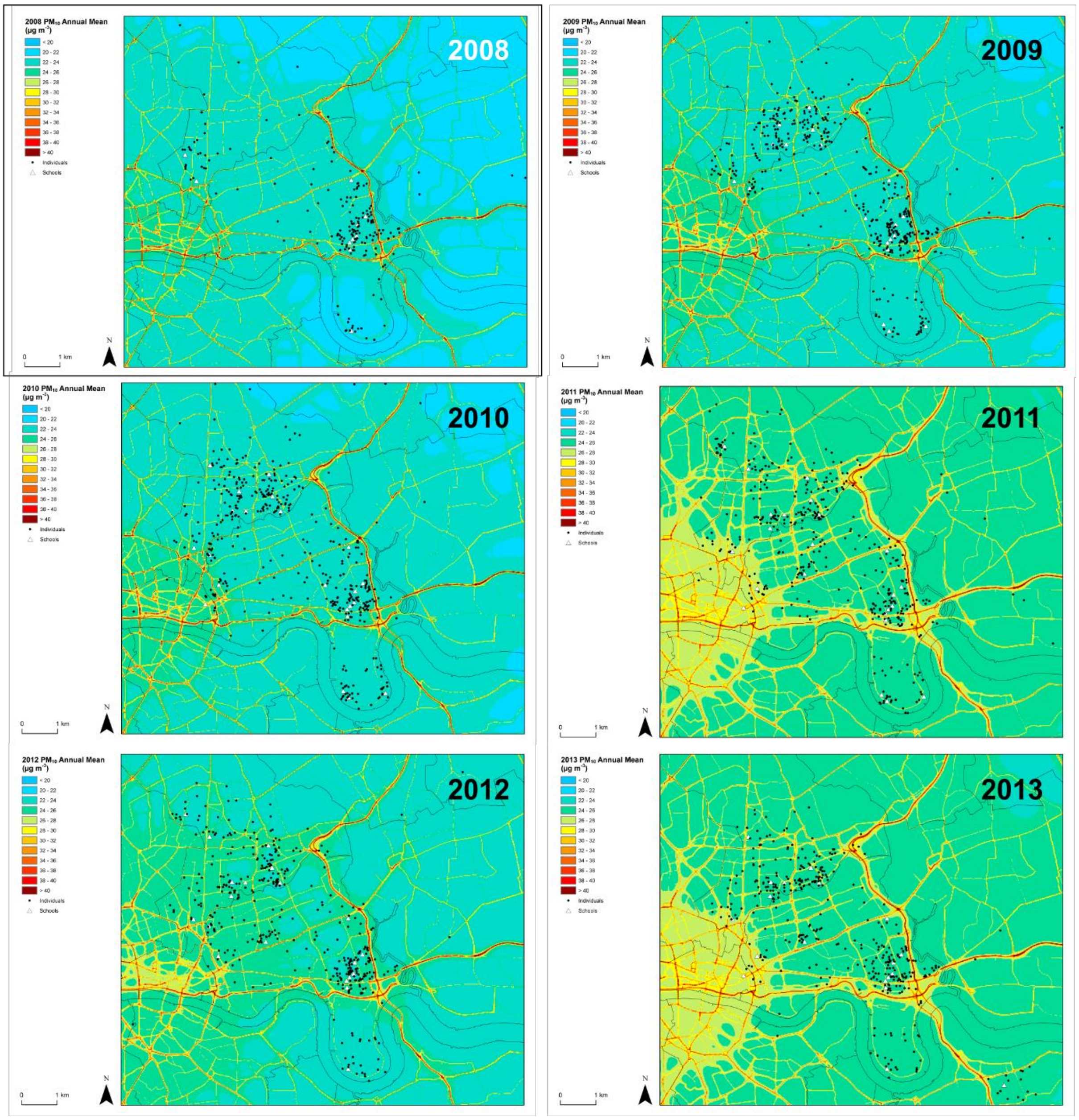

Figure s2: Annual PM_{10} maps for the years 2008 -2013.

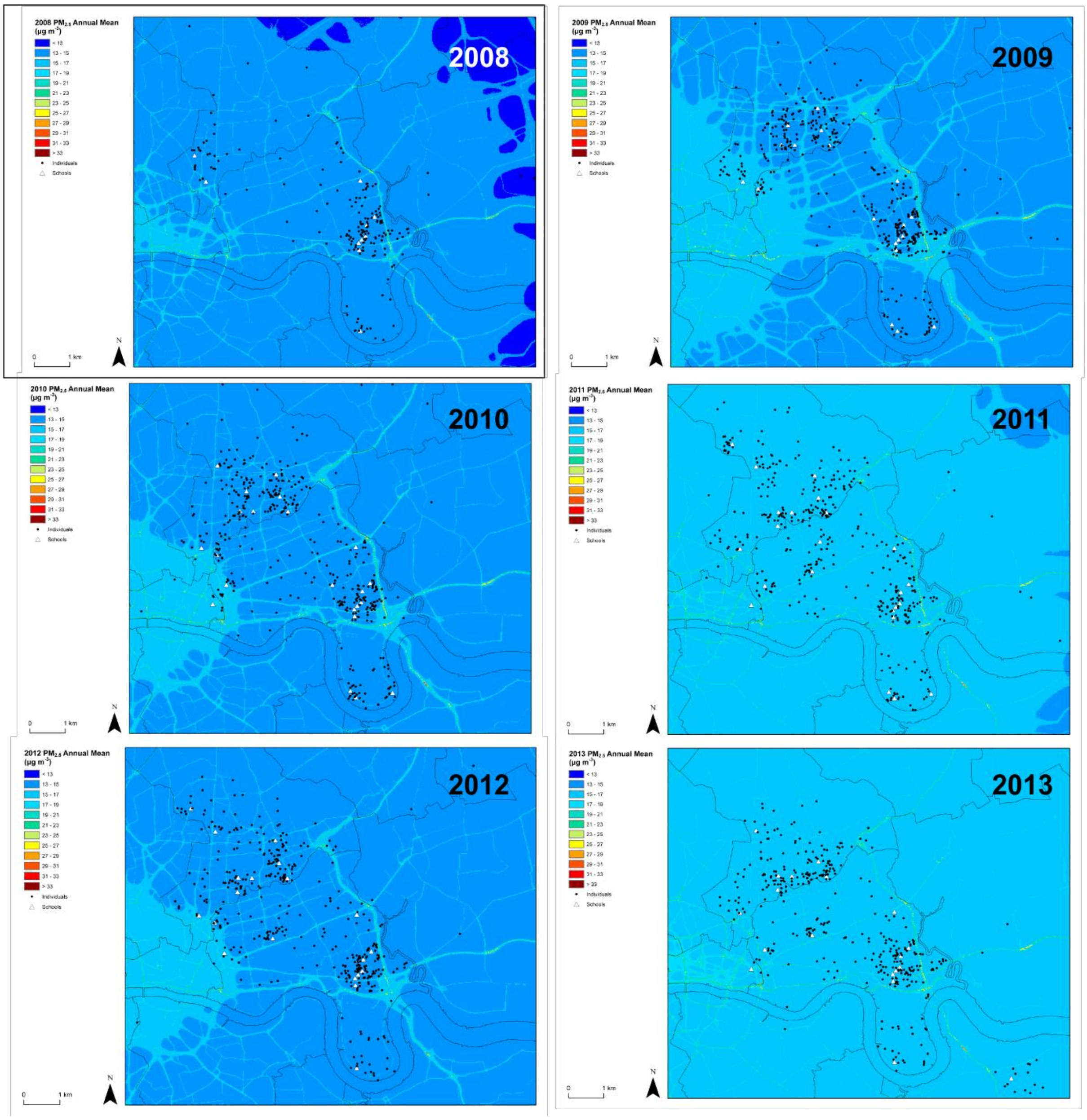

Figure s3: Annual $PM_{2.5}$ maps for the years 2008 -2013.

Table s15: Modelled annual pollutant attributions (Home and Home+School) by study year (μg/m^3 – median with 25th and 75th percentiles).

	**Pilot**<br>*08/09*<br>*N=199*	**Year 1**<br>*09/10*<br>*N=440*	**Year 2**<br>*10/11*<br>*N=418*	**Year 3**<br>*11/12*<br>*N=424*	**Year 4**<br>*12/13*<br>*N=423*	**Year 5**<br>*13/14*<br>*N=422*	**Year 1-5**<br>*09-14*<br>*N=2127*
**NOx** (H)	68.4<br>(64.4-74.0)	75.6<br>(73.1-81.9)	69.6<br>(66.6-76.3)	61.6<br>(58.8-65.5)	68.4<br>(65.5-72.5)	63.4<br>(60.8-68.3)	**68.4**<br>**(63.3-74.7)**
**NOx (**H+S)	71.4<br>(67.3-76.9)	78.8<br>(76.0-85.2)	73.3<br>(69.9-80.7)	64.3<br>(61.6-68.2)	71.3<br>(68.3-75.1)	66.1<br>(63.4-70.5)	**71.5**<br>**(65.9-78.1)**
**NO_2** (H)	41.0<br>(39.5-43.4)	43.5<br>(42.4-45.9)	41.2<br>(40.0-43.8)	38.0<br>(36.7-39.6)	40.8<br>(39.6-42.7)	38.8<br>(37.6-40.9)	**40.7**<br>**(38.7-43.3)**
**NO_2** (H+S)	42.6<br>(41.1-44.9)	45.3<br>(44.2-47.8)	43.1<br>(41.7-46.1)	39.6<br>(38.4-41.2)	42.5<br>(43.1-44.2)	40.4<br>(39.3-42.5)	**42.5**<br>**(40.3-45.1)**
**PM_{10}** (H)	22.6<br>(22.2-23.1)	23.5<br>(23.3-24.1)	22.6<br>(22.3-23.2)	25.6<br>(25.3-26.1)	24.2<br>(23.9-24.7)	25.4<br>(25.2-26.0)	**24.6**<br>**(23.4-25.5)**
**PM_{10}** (H+S)	23.5<br>(23.1-24.0)	24.5<br>(24.2-25.0)	23.6<br>(23.2-24.2)	26.6<br>(26.3-27.2)	25.2<br>(24.9-25.7)	26.4<br>(26.2-27.0)	**25.6**<br>**(24.4-26.5)**
**$PM_{2.5}$** (H)	12.9<br>(12.7-13.2)	14.3<br>(14.2-14.5)	13.2<br>(13.0-13.4)	15.7<br>(15.5-15.9)	14.4<br>(14.3-14.6)	16.0<br>(15.9-16.2)	**14.6**<br>**(14.2-15.8)**
**$PM_{2.5}$ (**H+S)	13.5<br>(13.2-13.7)	14.9<br>(14.7-15.1)	13.7<br>(13.6-14.0)	16.3<br>(16.2-16.5)	15.0<br>(14.9-15.2)	16.6<br>(16.5-16.8)	**15.2**<br>**(14.7-16.4)**

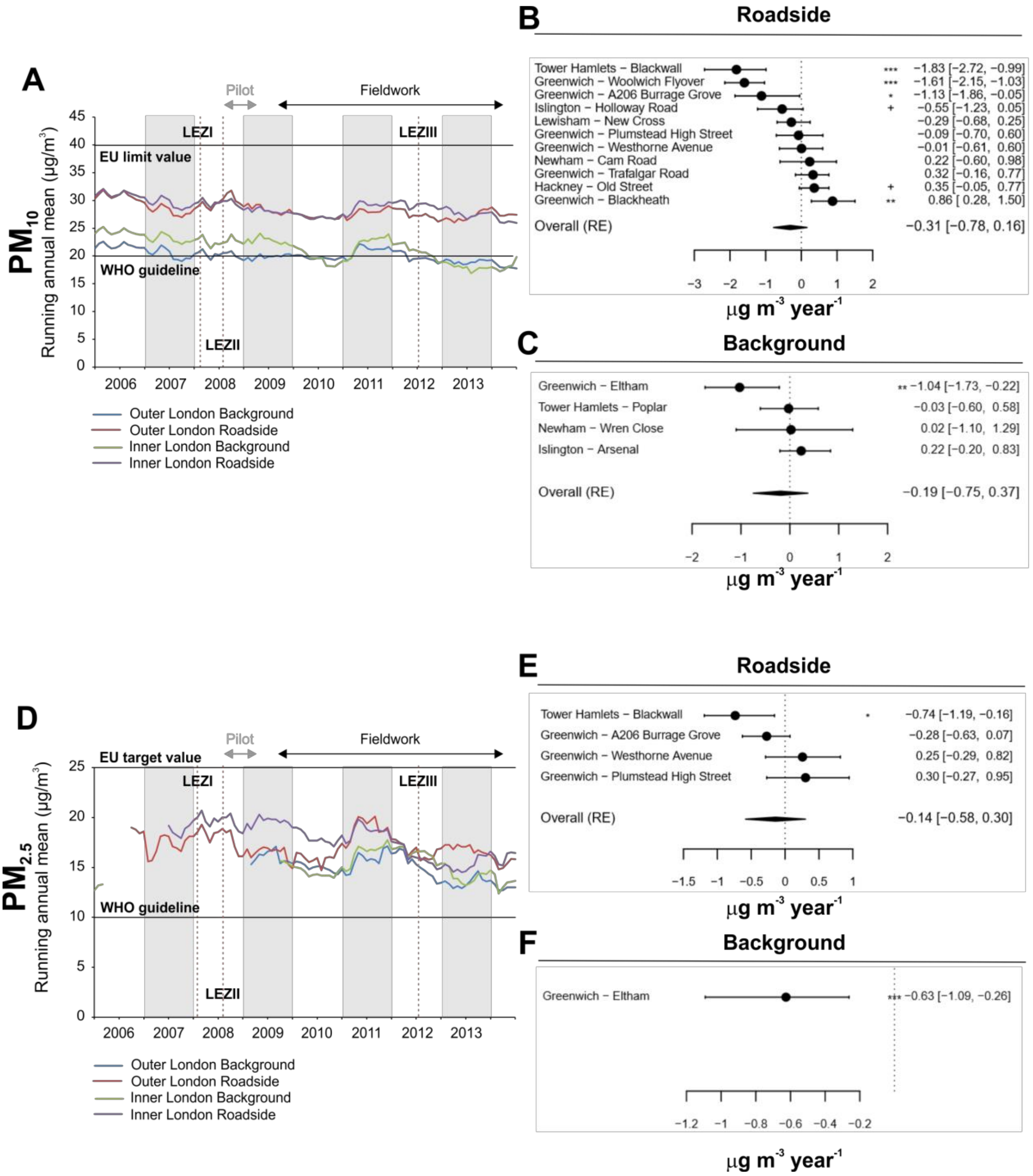

Figure s4: Running annual mean PM_{10} and $PM_{2.5}$ concentrations at inner and outer London roadside and background sites within and surrounding the study area from the beginning of 2006 to 2014 (panels A and D). Air pollution trends are shown relative the three phases of the LEZ. Panels B and C show Forest plots of roadside and background trends in PM_{10} (changes in air pollutant concentrations per year) across the period 2008-2013 by site and aggregated across sites. Panels E and F illustrate the equivalent data for $PM_{2.5}$. *** significance at the <0.001, ** significance at <0.01, * significance at <0.05, per site and aggregated across sites.

Table s16: Effects of air pollutants on post-bronchodilator FEV_1 in study years 1-5 and with the pilot year included.

Air pollutant Exposure		FEV_1 Adjusted models* Years 1-5			FEV_1 Adjusted models* Years 1-5 + pilot		
		N	Coeff, 95% CI	p	N	Coeff, 95% CI	p
Annual, home address	NOx	1900	-0.0005 [-0.0012,0.0002]	0.15	2030	-0.0005 [-0.0012,0.0002]	0.16
	NO_2	1900	-0.0013 [-0.0031,0.0006]	0.18	2030	-0.0012 [-0.0030,0.0006]	0.20
	PM_{10}	1900	-0.0052 [-0.0126,0.0022]	0.17	2030	-0.0048 [-0.0119,0.0022]	0.18
	$PM_{2.5}$	1900	-0.0109 [-0.0284,0.0065]	0.22	2030	-0.0103 [-0.0269,0.0064]	0.23
7days prior, home address	NOx	1870	0.0001 [-0.0005,0.0006]	0.84	2000	0.0000 [-0.0005,0.0005]	0.96
	NO_2	1870	0.0009 [-0.0008,0.0026]	0.31	2000	0.0006 [-0.0011,0.0022]	0.49
	**PM_{10}**	**1870**	**-0.0017 [-0.0029,-0.0005]**	**P<0.01**	**2000**	**-0.0018 [-0.0030,-0.0007]**	**P<0.01**
	**$PM_{2.5}$**	**1870**	**-0.0019 [-0.0035,-0.0004]**	**0.013**	**2000**	**-0.0021 [-0.0036,-0.0006]**	**P<0.01**
1day prior, home address	NOx	1870	0.0001 [-0.0001,0.0004]	0.32	2000	0.0001 [-0.0002,0.0003]	0.56
	NO_2	1870	0.0005 [-0.0005,0.0014]	0.33	2000	0.0003 [-0.0007,0.0012]	0.57
	PM_{10}	1870	0.0000 [-0.0008,0.0008]	0.99	2000	-0.0001 [-0.0009,0.0007]	0.81
	$PM_{2.5}$	1870	0.0003 [-0.0008,0.0013]	0.60	2000	0.0002 [-0.0008,0.0012]	0.69
6-9am prior, home address	NOx	1870	0.0002 [-0.0000,0.0003]	0.076	2000	0.0001 [-0.0000,0.0003]	0.17
	NO_2	1870	0.0006 [-0.0001,0.0014]	0.11	2000	0.0005 [-0.0002,0.0012]	0.19
	PM_{10}	1870	0.0005 [-0.0002,0.0011]	0.18	2000	0.0004 [-0.0002,0.0011]	0.22
	$PM_{2.5}$	1870	0.0007 [-0.0001,0.0015]	0.086	2000	0.0007 [-0.0001,0.0015]	0.10
Annual, home & school	NOx	1900	-0.0005 [-0.0013,0.0002]	0.19	2030	-0.0005 [-0.0012,0.0003]	0.20
	NO_2	1900	-0.0012 [-0.0032,0.0007]	0.22	2030	-0.0012 [-0.0031,0.0008]	0.24
	PM_{10}	1900	-0.005 [-0.0130,0.0030]	0.22	2030	-0.0047 [-0.0124,0.0030]	0.23
	$PM_{2.5}$	1900	-0.0101 [-0.0288,0.0085]	0.29	2030	-0.0097 [-0.0276,0.0082]	0.29
7days prior, home & school	NOx	1870	0.0001 [-0.0005,0.0006]	0.75	2000	0.0000 [-0.0005,0.0005]	0.96
	NO_2	1870	0.001 [-0.0007,0.0027]	0.24	2000	0.0007 [-0.0009,0.0023]	0.40
	**PM_{10}**	**1870**	**-0.0016 [-0.0028,-0.0005]**	**P<0.01**	**2000**	**-0.0018 [-0.0029,-0.0006]**	**P<0.01**
	**$PM_{2.5}$**	**1870**	**-0.0018 [-0.0033,-0.0004]**	**0.014**	**2000**	**-0.002 [-0.0035,-0.0005]**	**P<0.01**
1day prior, home & school	NOx	1870	0.0001 [-0.0001,0.0004]	0.28	2000	0.0001 [-0.0002,0.0003]	0.50
	NO_2	1870	0.0005 [-0.0004,0.0014]	0.28	2000	0.0003 [-0.0006,0.0012]	0.51
	PM_{10}	1870	0.0000 [-0.0007,0.0008]	0.95	2000	-0.0001 [-0.0008,0.0007]	0.85
	$PM_{2.5}$	1870	0.0003 [-0.0007,0.0013]	0.58	2000	0.0002 [-0.0008,0.0012]	0.67
6-9am prior, home & school	NOx	1870	0.0002 [-0.0000,0.0003]	0.061	2000	0.0001 [-0.0000,0.0003]	0.14
	NO_2	1870	0.0007 [-0.0001,0.0014]	0.086	2000	0.0005 [-0.0002,0.0012]	0.16
	PM_{10}	1870	0.0005 [-0.0002,0.0011]	0.16	2000	0.0004 [-0.0002,0.0010]	0.20
	$PM_{2.5}$	1870	0.0007 [-0.0001,0.0015]	0.081	2000	0.0007 [-0.0001,0.0014]	0.095

* Models are adjusted for age, gender, height, BMI, IMD deprivation index, urinary cotinine:creatine ratio >30, reported ethnicity and study year and includes a random intercept for school.

Table s17: Effects of air pollutants on post-bronchodilator FVC in study years 1-5 and with the pilot year included.

Air pollutant Exposure		FVC Adjusted models* Years 1-5			FVC Adjusted models* Years 1-5 + pilot		
		N	Coeff, 95% CI	p	N	Coeff, 95% CI	p
Annual, home address	**NOx**	**1859**	**-0.0009 [-0.0017,-0.0001]**	**0.026**	**1988**	**-0.0009 [-0.0017,-0.0001]**	**0.023**
	**NO_2**	**1859**	**-0.0023 [-0.0044,-0.0002]**	**0.033**	**1988**	**-0.0023 [-0.0043,-0.0002]**	**0.031**
	**PM_{10}**	**1859**	**-0.0090 [-0.0175,-0.0005]**	**0.038**	**1988**	**-0.0087 [-0.0168,-0.0006]**	**0.036**
	$PM_{2.5}$	1859	-0.0193 [-0.0392,0.0007]	0.058	1988	-0.0187 [-0.0378,0.0005]	0.056
7days prior, home address	NOx	1829	-0.0002 [-0.0008,0.0004]	0.53	1958	-0.0003 [-0.0009,0.0003]	0.32
	NO_2	1829	-3.7e-05 [-0.0020,0.0019]	0.97	1958	-0.0005 [-0.0024,0.0014]	0.63
	**PM_{10}**	**1829**	**-0.0017 [-0.0031,-0.0004]**	**0.013**	**1958**	**-0.002 [-0.0033,-0.0006]**	**P<0.01**
	**$PM_{2.5}$**	**1829**	**-0.0021 [-0.0038,-0.0003]**	**0.022**	**1958**	**-0.0023 [-0.0041,-0.0005]**	**0.010**
1day prior, home address	NOx	1829	0.0001 [-0.0002,0.0004]	0.69	1958	-6.0e-06 [-0.0003,0.0003]	0.97
	NO_2	1829	0.0002 [-0.0009,0.0013]	0.77	1958	-0.0001 [-0.0012,0.0010]	0.86
	PM_{10}	1829	-0.0001 [-0.0010,0.0008]	0.82	1958	-0.0002 [-0.0011,0.0006]	0.59
	$PM_{2.5}$	1829	2.9e-05 [-0.0012,0.0012]	0.96	1958	-0.0001 [-0.0013,0.0011]	0.90
6-9am prior, home address	NOx	1829	0.0002 [-0.0001,0.0004]	0.14	1958	0.0001 [-0.0001,0.0003]	0.29
	NO_2	1829	0.0006 [-0.0003,0.0015]	0.19	1958	0.0004 [-0.0004,0.0013]	0.34
	PM_{10}	1829	0.0004 [-0.0003,0.0012]	0.28	1958	0.0003 [-0.0004,0.0011]	0.40
	$PM_{2.5}$	1829	0.0007 [-0.0002,0.0017]	0.13	1958	0.0006 [-0.0003,0.0016]	0.18
Annual, home & school	**NOx**	**1859**	**-0.0009 [-0.0018,-0.0001]**	**0.033**	**1988**	**-0.001 [-0.0018,-0.0001]**	**0.028**
	**NO_2**	**1859**	**-0.0023 [-0.0046,-0.0001]**	**0.042**	**1988**	**-0.0023 [-0.0046,-0.0001]**	**0.037**
	PM_{10}	1859	-0.0091 [-0.0183,-0.0000]	0.050	**1988**	**-0.009 [-0.0178,-0.0002]**	**0.044**
	$PM_{2.5}$	1859	-0.0189 [-0.0401,0.0023]	0.080	1988	-0.0188 [-0.0393,0.0017]	0.073
7days prior, home & school	NOx	1829	-0.0002 [-0.0008,0.0005]	0.61	1958	-0.0003 [-0.0009,0.0003]	0.37
	NO_2	1829	0.0001 [-0.0018,0.0020]	0.91	1958	-0.0003 [-0.0022,0.0016]	0.73
	**PM_{10}**	**1829**	**-0.0016 [-0.0030,-0.0003]**	**0.015**	**1958**	**-0.0019 [-0.0032,-0.0006]**	**P<0.01**
	**$PM_{2.5}$**	**1829**	**-0.002 [-0.0036,-0.0003]**	**0.023**	**1958**	**-0.0022 [-0.0039,-0.0005]**	**0.011**
1day prior, home & school	NOx	1829	0.0001 [-0.0002,0.0004]	0.63	1958	6.0e-06 [-0.0003,0.0003]	0.97
	NO_2	1829	0.0002 [-0.0009,0.0013]	0.70	1958	-0.0001 [-0.0011,0.0010]	0.927
	PM_{10}	1829	-0.0001 [-0.0009,0.0008]	0.86	1958	-0.0002 [-0.0011,0.0006]	0.62
	$PM_{2.5}$	1829	4.2e-05 [-0.0011,0.0012]	0.94	1958	-0.0001 [-0.0012,0.0011]	0.92
6-9am prior, home & school	NOx	1829	0.0002 [-0.0000,0.0004]	0.12	1958	0.0001 [-0.0001,0.0003]	0.25
	NO_2	1829	0.0006 [-0.0002,0.0015]	0.16	1958	0.0004 [-0.0004,0.0013]	0.30
	PM_{10}	1829	0.0004 [-0.0003,0.0012]	0.26	1958	0.0003 [-0.0004,0.0011]	0.37
	$PM_{2.5}$	1829	0.0007 [-0.0002,0.0016]	0.13	1958	0.0006 [-0.0003,0.0015]	0.17

* Models are adjusted for age, gender, height, BMI, IMD deprivation index, urinary cotinine:creatine ratio >30, reported ethnicity and study year and includes a random intercept for school.

Table s18: Effects of air pollutants on post-bronchodilator FEV_1 and FVC in study years 1-5. Data are expressed as a change in volume (L) per IQR of modelled pollutant concentrations at residential address and weighted for the period spent at school.

**Air pollutant Exposure**		**FEV_1 Adjusted models*** Years 1-5			**FVC Adjusted models*** **Years 1-5**		
		**N**	**Coeff, 95% CI**	**p**	**N**	**Coeff, 95% CI**	**p**
Annual, home address	NOx	1900	-0.006 [-0.015,0.002]	0.15	**1859**	**-0.011 [-0.021,-0.001]**	**0.026**
	NO_2	1900	-0.006 [-0.015,0.003]	0.18	**1859**	**-0.0011 [-0.021,-0.001]**	**0.033**
	PM_{10}	1900	-0.011 [-0.026,0.004]	0.17	**1859**	**-0.019 [-0.036,-0.001]**	**0.038**
	$PM_{2.5}$	1900	-0.018 [-0.047,0.011]	0.22	1859	-0.032 [-0.065,0.001]	0.058
Annual, home & school	NOx	1900	-0.006 [-0.015,0.002]	0.19	**1859**	**-0.010 [-0.021,-0.001]**	**0.033**
	NO_2	1900	-0.006 [-0.015,0.003]	0.22	**1859**	**-0.011 [-0.021,0.000]**	**0.042**
	PM_{10}	1900	-0.010 [-0.026,0.006]	0.22	1859	-0.018 [-0.037,-0.000]	0.050
	$PM_{2.5}$	1900	-0.016 [-0.046,0.014]	0.29	1859	-0.030 [-0.064,0.004]	0.080

Pollutant interquartile ranges over the five-year study period for the individuals with spirometry meeting the inclusion criteria were as follows. For exposure attributions at residential address (home): NOx, 12.2; NO_2, 4.8; PM_{10}, 2.1; $PM_{2.5}$, 1.7 $\mu g/m^3$. For exposure attributions weighted for periods spent at home and school: NOx, 11.5; NO_2, 4.6; PM_{10}, 2.0; $PM_{2.5}$, 1.6 $\mu g/m^3$.

Table s19: Effects of air pollutants on pre-bronchodilator FEV_1 and FVC in study years 1-5

Air pollutant exposure		FEV_1 Adjusted models* Years 1-5			FVC Adjusted models* Years 1-5		
		N	Coeff, 95% CI	p	N	Coeff, 95% CI	p
Annual, home address	NOx	1908	-0.0004 [-0.0011,0.0003]	0.30	1876	-0.0008 [-0.0016,0.0001]	0.069
	NO_2	1908	-0.0009 [-0.0028,0.0009]	0.32	1876	-0.0019 [-0.0040,0.0002]	0.070
	PM_{10}	1908	-0.0045 [-0.0120,0.0030]	0.24	1876	-0.0084 [-0.0169,0.0000]	0.050
	$PM_{2.5}$	1908	-0.0097 [-0.0273,0.0079]	0.28	1876	-0.0182 [-0.0381,0.0016]	0.072
7days prior, home address	NOx	1875	-0.0001 [-0.0006,0.0005]	0.83	1844	-0.0001 [-0.0007,0.0006]	0.85
	NO_2	1875	0.0005 [-0.0012,0.0022]	0.59	1844	0.0003 [-0.0016,0.0022]	0.76
	**PM_{10}**	**1875**	**-0.0017 [-0.0030,-0.0005]**	**P<0.01**	**1844**	**-0.0017 [-0.0030,-0.0003]**	**0.018**
	**$PM_{2.5}$**	**1875**	**-0.0022 [-0.0037,-0.0007]**	**P<0.01**	**1844**	**-0.0019 [-0.0037,-0.0001]**	**0.034**
1day prior, home address	NOx	1875	0 [-0.0003,0.0003]	0.91	1844	4.0e-05 [-0.0003,0.0003]	0.80
	NO_2	1875	0.0004 [-0.0006,0.0013]	0.46	1844	0.0003 [-0.0008,0.0014]	0.59
	PM_{10}	1875	-0.0003 [-0.0011,0.0004]	0.40	1844	-0.0002 [-0.0011,0.0007]	0.66
	$PM_{2.5}$	1875	-0.0003 [-0.0013,0.0008]	0.61	1844	-0.0002 [-0.0013,0.0010]	0.79
6-9am prior, home address	NOx	1875	0.0001 [-0.0001,0.0003]	0.32	1844	0.0001 [-0.0001,0.0003]	0.28
	NO_2	1875	0.0005 [-0.0002,0.0013]	0.17	1844	0.0005 [-0.0004,0.0014]	0.26
	PM_{10}	1875	0.0001 [-0.0006,0.0008]	0.74	1844	0.0003 [-0.0005,0.0010]	0.51
	$PM_{2.5}$	1875	0.0003 [-0.0005,0.0011]	0.47	1844	0.0005 [-0.0005,0.0014]	0.35
Annual, home & school	NOx	1908	-0.0004 [-0.0011,0.0004]	0.37	1876	-0.0008 [-0.0016,0.0001]	0.086
	NO_2	1908	-0.0009 [-0.0028,0.0011]	0.39	1876	-0.002 [-0.0042,0.0003]	0.088
	PM_{10}	1908	-0.0042 [-0.0123,0.0038]	0.30	1876	-0.0087 [-0.0178,0.0005]	0.063
	$PM_{2.5}$	1908	-0.0087 [-0.0275,0.0101]	0.36	1876	-0.0182 [-0.0394,0.0031]	0.094
7days prior, home & school	NOx	1875	0.0000 [-0.0006,0.0005]	0.90	1844	-2.4e-05 [-0.0007,0.0006]	0.94
	NO_2	1875	0.0006 [-0.0011,0.0023]	0.50	1844	0.0005 [-0.0015,0.0024]	0.64
	**PM_{10}**	**1875**	**-0.0017 [-0.0028,-0.0005]**	**P<0.01**	**1844**	**-0.0016 [-0.0029,-0.0002]**	**0.021**
	**$PM_{2.5}$**	**1875**	**-0.0021 [-0.0036,-0.0006]**	**P<0.01**	**1844**	**-0.0018 [-0.0035,-0.0001]**	**0.036**
1day prior, home & school	NOx	1875	0.0000 [-0.0002,0.0003]	0.86	1844	4.9e-05 [-0.0003,0.0003]	0.75
	NO_2	1875	0.0004 [-0.0006,0.0013]	0.42	1844	0.0003 [-0.0007,0.0014]	0.53
	PM_{10}	1875	-0.0003 [-0.0011,0.0004]	0.42	1844	-0.0002 [-0.0010,0.0007]	0.69
	$PM_{2.5}$	1875	-0.0003 [-0.0013,0.0008]	0.62	1844	-0.0001 [-0.0013,0.0010]	0.81
6-9am prior, home & school	NOx	1875	0.0001 [-0.0001,0.0003]	0.28	1844	0.0001 [-0.0001,0.0003]	0.25
	NO_2	1875	0.0006 [-0.0002,0.0013]	0.15	1844	0.0005 [-0.0003,0.0014]	0.22
	PM_{10}	1875	0.0001 [-0.0005,0.0008]	0.71	1844	0.0003 [-0.0005,0.0010]	0.48
	$PM_{2.5}$	1875	0.0003 [-0.0005,0.0011]	0.46	1844	0.0004 [-0.0005,0.0014]	0.34

* Models are adjusted for age, gender, height, BMI, IMD deprivation index, urinary cotinine:creatine ratio >30, reported ethnicity and study year and includes a random intercept for school.

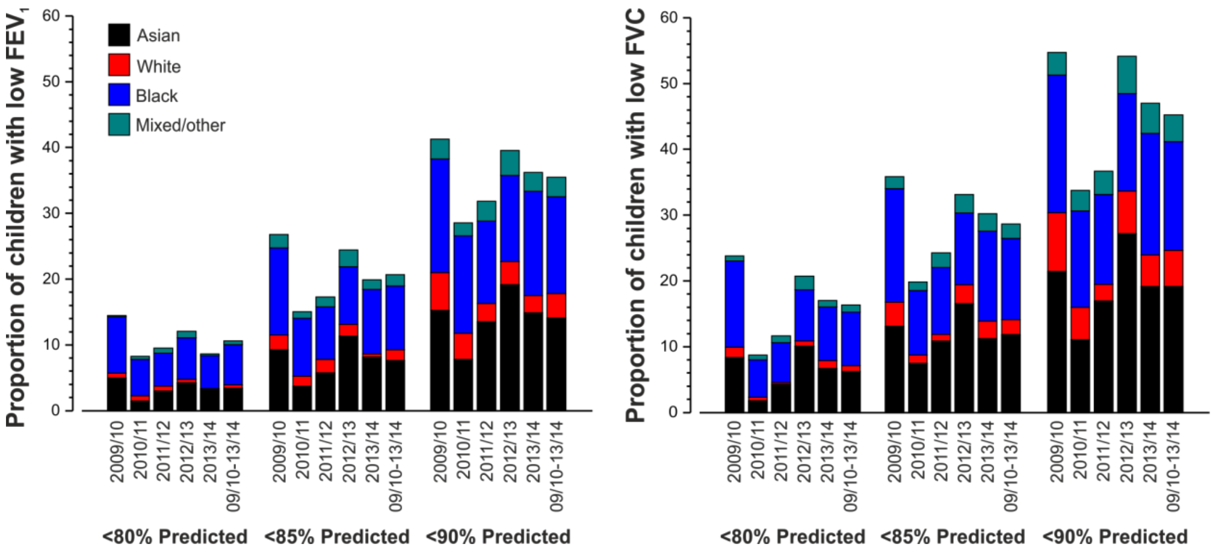

Figure s5: Proportion of children with lung function (FEV_1 and FVC) below 90, 85 and 80% of predicted. Contributions of the 4-main ethnic groups are shown.

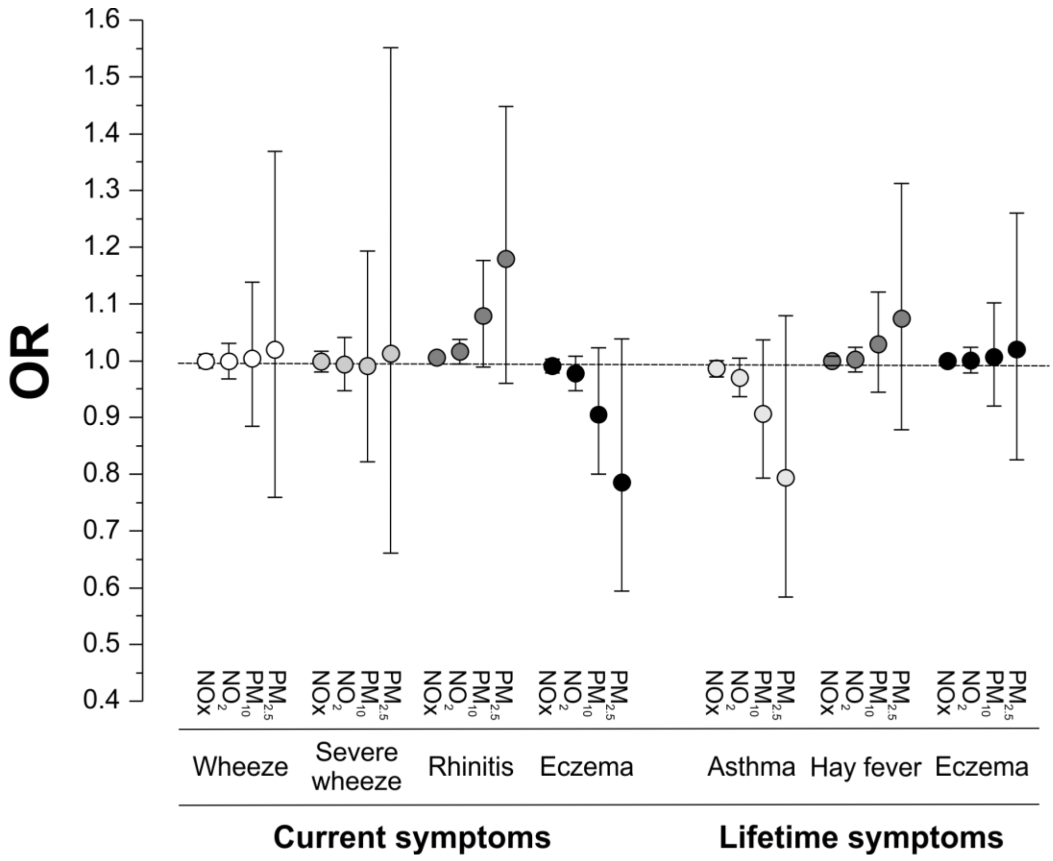

Figure s6: Odds ratios and 95% confidence intervals for current and lifetime allergic and respiratory symptoms in relation to annual pollutant exposure attributions based on residential address.

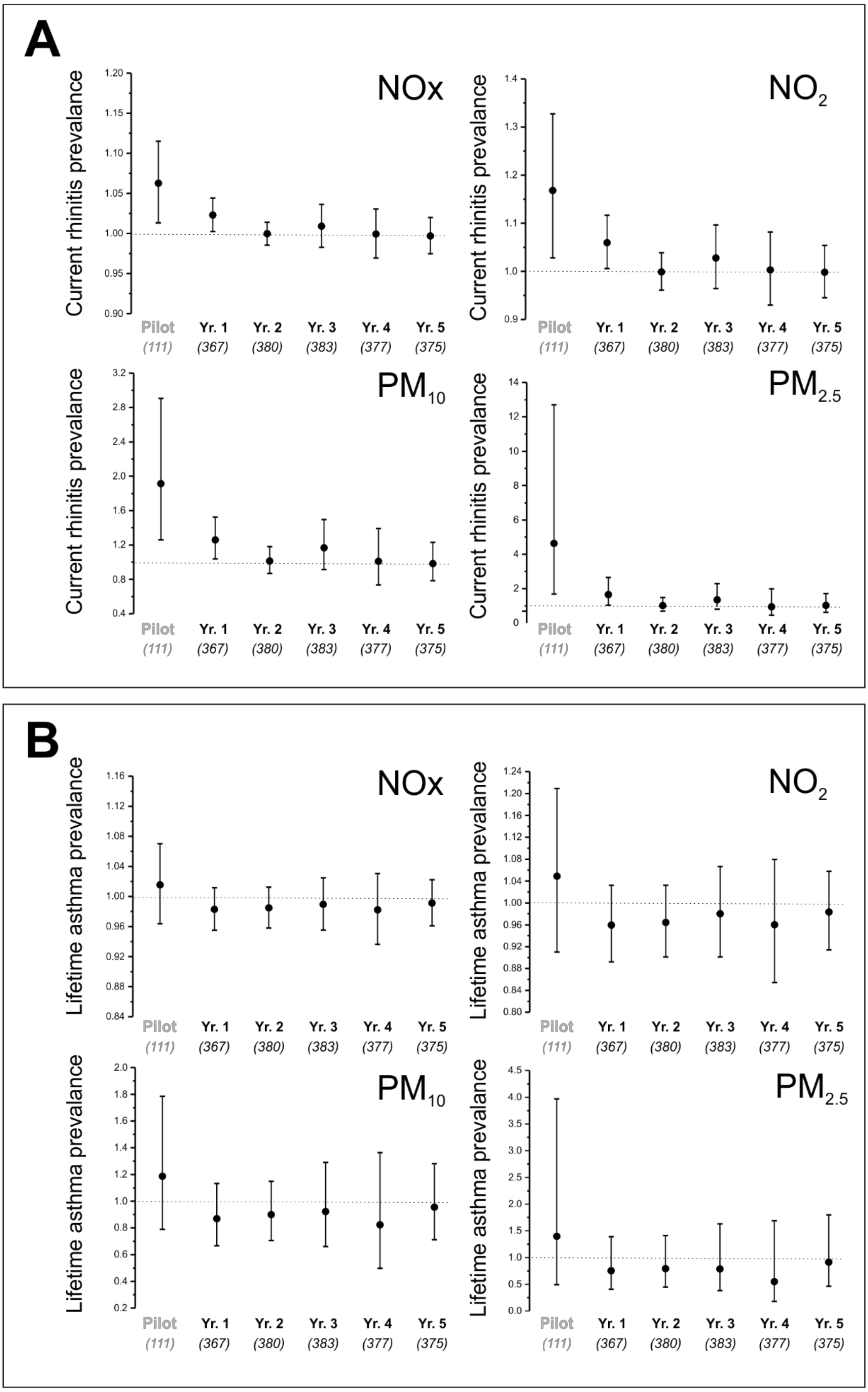

Figure s7: Yearly prevalence of rhinitis (panel A) and lifetime asthma (panel B) in relation to annual exposure to NOx, NO_2, PM_{10} and $PM_{2.5}$, based on residential address.

Supplementary References

S1 Transport of London. Transport and air quality strategy revisions: London Low Emission Zone, supplementary material, public and stakeholder consultation. 2006. http://content.tfl.gov.uk/supplementary-info.pdf (accessed 20th March 2017)

S2 Holman, CH, Harrison, R, and Querol, X. Review of the efficacy of low emission zones to improve urban air quality in European cities. Atmospheric Environment. 2015; **111**:161-9.

S3 Greater London Authority (GLA). The London Atmospheric Emissions Inventory 2008 http://data.london.gov.uk/laei-2008, 2010. Accessed 31/05/2011

S4 Greater London Authority (GLA). The London Atmospheric Emissions Inventory 2008 http://data.london.gov.uk/laei-2008, 2010. Accessed 31/05/2011

S5 AMDS-Roads - An Air Quality Management System User Guide. Cambridge Environmental Research Consultants Ltd, Cambridgehttp://www.cerc.co.uk/environmental-software/assets/data/doc_userguides/CERC_ADMS-Roads%20Extra3.1_User_Guide.pdf (accessed 03 January 2012), 2006.

S6 Beevers, SD, Kitwiroon, N, Williams, ML, et al. Air pollution dispersion models for human exposure predictions in London. *Journal of exposure science & environmental epidemiology.* 2013; **23**(6):647-53.

S7 Carslaw, DC. Defra Urban Model Evaluation Analysis – Phase 1. http://uk-air.defra.gov.uk/library/reports?report_id=654. 2011.

S8 Dons, E, Van Poppel, M, Int Panis, L. et al. Land use regression models as a tool for short, medium and long-term exposure to traffic related air pollution. *Sci Total Environ*. 2014;**476-477**:378-86.

S9 Johnson, M, Macneill, M, Grgicak-Mannion A, et al. Development of temporally refined land-use regression models predicting daily household-level air pollution in a panel study of lung function among asthmatic children. J Expo Sci Environ Epidemiol. 2013;**23**(3):259-67.

S10 Derwent, R, Fraser, A, Abbott, J, et al. Evaluating the Performance of Air Quality Models. 2010. www.airquality.co.uk/reports/cat05/1006241607_100608_MIP_Final_Version.pdf.

S11 Miller, MR, Hankinson, J, Brusasco, V, et al. Standardisation of spirometry. *Eur Respir J.* 2005; **26**(2):319-38.

S12 Praud, JP. Snoring in children: still many questions, only a few answers. *Pediatr Pulmonol Suppl.* 2004; **26**:169-71**.**

S13 Stanojevic S, Wade A, Cole TJ, et al. Spirometry centile charts for young Caucasian children: the Asthma UK Collaborative Initiative. *Am J Respir Crit Care Med* 2009;**180**(6):547-52.
